# Fractalkine isoforms differentially regulate microglia-mediated inflammation and enhance visual function in the diabetic retina

**DOI:** 10.1186/s12974-023-02983-8

**Published:** 2024-02-04

**Authors:** Derek Rodriguez, Kaira A. Church, Alicia N. Pietramale, Sandra M. Cardona, Difernando Vanegas, Colin Rorex, Micah C. Leary, Isabel A. Muzzio, Kevin R. Nash, Astrid E. Cardona

**Affiliations:** 1 Department of Molecular Microbiology and Immunology, UTSA Circle, The University of Texas at San Antonio, San Antonio, TX 78249 USA; 2 Department of Psychological and Brain Sciences, University of Iowa, Iowa City, IA 52242 USA; 3 Department of Molecular Pharmacology and Physiology, University of South Florida, Tampa, FL 33620 USA

**Keywords:** Microglia, Macrophages, Fractalkine, Diabetes, Gene therapy, Inflammation

## Abstract

Diabetic retinopathy (DR) affects about 200 million people worldwide, causing leakage of blood components into retinal tissues, leading to activation of microglia, the resident phagocytes of the retina, promoting neuronal and vascular damage. The microglial receptor, CX3CR1, binds to fractalkine (FKN), an anti-inflammatory chemokine that is expressed on neuronal membranes (mFKN), and undergoes constitutive cleavage to release a soluble domain (sFKN). Deficiencies in CX3CR1 or FKN showed increased microglial activation, inflammation, vascular damage, and neuronal loss in experimental mouse models. To understand the mechanism that regulates microglia function, recombinant adeno-associated viral vectors (rAAV) expressing mFKN or sFKN were delivered to intact retinas prior to diabetes. High-resolution confocal imaging and mRNA-seq were used to analyze microglia morphology and markers of expression, neuronal and vascular health, and inflammatory mediators. We confirmed that prophylactic intra-vitreal administration of rAAV expressing sFKN (rAAV–sFKN), but not mFKN (rAAV–mFKN), in FKN^KO^ retinas provided vasculo- and neuro-protection, reduced microgliosis, mitigated inflammation, improved overall optic nerve health by regulating microglia-mediated inflammation, and prevented fibrin(ogen) leakage at 4 weeks and 10 weeks of diabetes induction. Moreover, administration of sFKN improved visual acuity. Our results elucidated a novel intervention via sFKN gene therapy that provides an alternative pathway to implement translational and therapeutic approaches, preventing diabetes-associated blindness.

**Supplementary Information:**

The online version contains supplementary material available at 10.1186/s12974-023-02983-8.

## Background

Diabetic retinopathy (DR), a common and severe complication of diabetes, leads to vision impairment and ocular morbidity in roughly 200 million patients globally [1]. Our group and others have shown that metabolic disease, partly due to the release of lipopolysaccharide (LPS), which induces low-grade systemic inflammation, influenced the severity of diabetes, [2–5] and exacerbated retinal damage in experimental DR [4]. Other studies suggest that high serum endotoxemia precedes the development of type 2 diabetes [6, 7]. The findings that diabetic patients present higher levels of serum endotoxin when compared to healthy individuals pose unanswered questions regarding the impact of this complex chronic inflammatory phenomena on disease progression [8]. It is unclear how recurrent episodes of systemic inflammation due to infections common in diabetic patients affect the progression and development of DR. Therefore, we aim to mimic the effects of systemic infection on retinal pathology commonly observed in diabetic patients.

DR is characterized by inflammation, microgliosis, neuronal damage, and abnormal changes in the blood vessels in the retina, which cause leakage of blood molecules into the retina, swelling, and the growth of new blood vessels [9, 10]. While the exact mechanisms underlying the development and progression of DR are still being elucidated, research has shed light on the involvement of microglia hyperactivation. Microglia, the resident professional phagocytes of the central nervous system (CNS), maintain homeostasis in the microenvironment of the retina, eliminating immunogens and promoting repair through their receptor, CX3CR1. Human polymorphic variants of the CX3CR1 gene encode a protein with amino acid substitutions at positions 249 (valine substituted for isoleucine) and 280 (threonine substituted for methionine), hCX3CR1^I249/M280^, in about 25% of the population [11, 12]. These mutations yield an adhesive–defective receptor with decreased binding affinity for its sole ligand, fractalkine (FKN or CX3CL1), promoting cellular stress and downstream vascular injury, neuronal cell death, and further amplifies neuroinflammation in DR [13, 14].

FKN is a unique chemokine and type I transmembrane glycoprotein composed of an N-terminal chemokine domain attached to a mucin-like stalk, followed by the transmembrane region, and intracellularly tapering a short C-terminal fragment. The membrane-bound (mFKN) protein is expressed predominantly on neuronal and endothelial surfaces, promoting cell adhesion and recruitment of infiltrating leukocytes to endothelial cells. Cleavage of mFKN occurs by proteases known as “sheddases” in a constitutively and induced manner to produce a soluble form (sFKN), involved in cell migration and vascular remodeling [15, 16]. FKN post-translational modifications are mediated primarily by A Disintegrin Metalloproteinase (ADAM) 10/17, TNF-α converting enzyme (TACE) and cathepsin S. [15, 17, 18]. Both mFKN and sFKN pose unique effects signaling through microglial CX3CR1 in many neurodegenerative diseases [19–21] and several studies support the contribution of the CX3CR1/FKN signaling axis in DR, but their biological activities in DR are still being elucidated. Ins2^Akita^ and streptozotocin (STZ) induced diabetes models, associated with insulin misfolding or deficient pancreatic β-islet cells, respectively, showed that in absence of CX3CR1 (CX3CR1^KO^), retinas displayed enhanced microglia activation, neuronal loss, and astrogliosis [2, 4, 22]. Lack of CX3CR1 also correlated with enhanced vascular damage, elevated NOS2 and IL-1β production [2]. Diabetic fractalkine knockout (FKN^KO^) mice showed enhanced fibrin(ogen) extravasation onto the parenchyma of the retina and perivascular clustering of microglia, mirroring the phenotype observed in the CX3CR1^KO^ mice. The administration of recombinant sFKN to FKN^KO^ retinas was shown to be effective in STZ murine models in mitigating microglial clustering and fibrin(ogen) deposition in the retina [4]. Moreover, microglia depletion and repopulation using PLX-5622 treatment ameliorated neuronal and vascular damage in the CX3CR1^WT^ microglia during DR. In contrast, repopulation of CX3CR1^KO^ or hCX3CR1^I249/M280^ microglia conferred worse retinal pathology in STZ models of diabetes. These data, supports the rationale that CX3CR1/FKN signaling axis is an important inhibitor of microglia hyperinflammation [3, 23]. Therefore, this study aims to understand the contribution of mFKN and sFKN in inflammation and vascular damage in the diabetic retina.

To understand the mechanisms by which FKN exerts neuroprotection in the diabetic retina, recombinant adeno-associated viruses (rAAV) were used to compare the effect of FKN isoforms in regulating microglia activation, vascular and neuronal damage, and visual function prior to the induction of diabetes. We show that FKN^KO^ mice, compared to WT mice, exhibited increased activation of microglia in the retina, brain, and spinal cord. Administration of rAAV–sFKN in FKN^KO^ mice was sufficient to restore microglia morphology to a physiological state in the CNS without altering the peripheral blood or secondary lymphoid organ immune environments. In addition, rAAV–sFKN prevented astrogliosis and axonal loss, while elevated presynaptic glycoprotein synaptophysin immunoreactivity in optic nerves. These findings indicate that neuroinflammation and vascular damage in the retina can be mitigated through novel molecular pathways in response to FKN gene therapy, providing an additional clinical option to stop retinal damage and improve visual acuity.

## Methods

### Mice

*CX3CL1*^*–/–*^ (referred to as FKN^KO^) mice were obtained from Dr. Sergio Lira (Icahn School of Medicine at Mount Sinai) and were bred and maintained at the laboratory animal resource core facility at the University of Texas at San Antonio (UTSA). All experiments used male mice 4–10 weeks. C57BL/6 wild-type (WT) (JAX stock number: 000664; RRID: IMSR_JAX:000664) mice were purchased from The Jackson Laboratory. Male mice, 4–10 weeks of age, were used for all experiments. Experiments were conducted according to the National Institutes of Health guidelines approved by the UTSA–Institutional Animal Care and Use Committee.

### Recombinant adeno-associated viral (rAAV) vector production

The vector constructs were engineered, assembled and produced by Dr. Kevin R. Nash (University of South Florida, Tampa, FL). rAAV serotype 9 vectors, flanked with AAV2 terminal repeat DNA sequences, expressing either mFKN or sFKN were subjected for cloning using PCR on cDNA isolated from mouse brain [24]. rAAV packing of sFKN protein expressed amino acids 1–336. rAAV packing of mFKN DNA comprises all 395 amino acids of full-length FKN protein and contained two mutations (R337A and R338A), preventing cleavage by proteases ADAM10/17 into the soluble form. Vectors of sFKN and mFKN were tagged with hemagglutinin (HA) at the C-terminus for feasible detection of exogenous protein. rAAV particles were quantified using a modified dot plot protocol and are expressed as vector genomes (vg)/mL as described [20].

### Intra-vitreal administration of rAAV

Mice were anesthetized with 5% isoflurane in oxygen. Prior to injections, the right palpebral was disinfected with a 70% alcohol prep pad (VWR, catalog number: 75856-902). Using a dissecting microscope and a 31G beveled Nanofil needle and syringe attached to a Micro 4TM Microsyringe Pump Controller (World Precision Instruments), the animal received a single intra-vitreal injection (200 nL/s for a total of 5 s) of rAAV expressing mFKN or sFKN at a concentration of 1 × 10^12^ vg/mL, diluted in 1 × PBS (Cytiva, catalog number: SH30258.02) in the right eye. The syringe was left in place for 5 s after the infusion to limit backflow, then removed slowly. One drop of Proparacaine Hydrochloride Ophthalmic Solution USP, 0.5% (Alcon, catalog number: H14233-0216) was applied to the eye as a topical antibiotic.

### Two-hit inflammatory streptozotocin (STZ)-induced model

Mice were injected intra-peritoneally (i.p.) with STZ, 60 mg/kg/day in citrate buffer for 5 days (Sigma-Aldrich, catalog number: S0130). Citrate buffer was used in non-diabetic (ND) mice, serving as controls. Blood glucose was monitored weekly after STZ injections for a duration of 4 weeks (4 wks) (onset) and 10 weeks (10 wks) (early) diabetes. Prior to end point, mice were i.p injected with LPS (*E. coli* serotype 055:B5; Sigma-Aldrich, catalog number: L2637), 1 mg/kg/day for 2 days [3]. PBS injected mice were used as controls. Mice were euthanized 4 h after final LPS injection.

### Tissue isolation for protein analyses

Mice were transcardially perfused with cold 1 × Hanks’ Balance Solution (HBSS; Cytiva, catalog number: SH30588.02).. To obtain total retinal protein, eyes were enucleated and dounce homogenized in lysis buffer prior to centrifugation at 12,000 rpm for 15 min at 4 °C to create a single cell suspension. The supernatant was collected and lysis buffer (ddH2O, 2.5 M NaCl, 1 M Tris, 0.5 M EDTA, and 100 × Protease Inhibitor; Sigma-Aldrich, catalog number: 04693116001) was added to the pellet prior to sonication. Samples were centrifuged at 12,000 rpm for 15 min at 4 °C, and supernatant that contained total retinal protein was collected and stored at − 80 °C until further analysis.

### Immunofluorescent staining

For tissue staining, retinal flat mounts were generated. For this, eyes were enucleated after perfusion and fixed in 4% buffered paraformaldehyde (PFA) (Sigma-Aldrich, catalog number: P6148) for 20 min at room temperature. The retina and optic nerves were isolated and fixed in 1% buffered PFA for 1 h. Dissected retinas were placed in cryoprotection solution (200 mL glycerol, 200 mL 0.4 M Sorenson’s buffer, and 600 mL MilliQ water) overnight at 4 °C, and then stored in cryostorage solution (500 mL 0.2 M PO4, 10 g PVP-40, 300 g sucrose, and 300 mL ethylene glycol) at − 20 °C until further analysis. For immunohistochemical staining, retinal flat mounts were blocked and permeabilized overnight at 4 °C with 1% Triton-X 100 in 10% Normal Goat Serum (Jackson ImmunoResearch Laboratories, RRID: AB_2336990). Tissues were incubated with primary antibodies overnight at 4 °C, followed by 7 washes for 5 min each of 0.1% Triton-X in 1 × PBS (Additional file 1: Table S1). Tissues were incubated in species-specific primary antibodies to visualize proteins of interest: rabbit anti-ionized calcium binding adaptor molecule-1 (Iba1), mouse anti-neuronal nuclei (NeuN), rabbit anti-synaptophysin (SYP), mouse anti-β tubulin III (TUJ1), rat anti-glial fibrillary acidic protein (GFAP), rat anti-pecam-1 (CD31), and rabbit anti-fibrinogen. Tissues were incubated in species-specific secondary antibodies, followed by Hoechst staining and mounted on slides using FluorSave reagent (Calbiochem, catalog number: 34578920ML). Combination of antibodies used for this paper are outlined in Additional file 1: Table S2.

### Fractalkine enzyme-linked immunosorbent assay

Protein quantification was performed using a Bradford assay. Standard Bovine Serum Albumin (BSA) (Bio-Rad, catalog number: 500-0007) was used as a standard at a 1.41 mg/mL concentration and serially diluted with 1 × HBSS. Retinal extracts were subjected to 1:2 dilution in 1 × HBSS. Protein dye (Bio-Rad, catalog number: 500-0006) was diluted to a 1:4 with ddH_2_O subjected to filtration using filer paper (ThermoFisher Scientific, catalog number: 1004110). Samples were plated as technical duplicates and read in a spectrophotometer with an absorbance of 595 nm. For ELISAs retinal protein isolates were normalized to concentration of 0.1 mg/mL. FKN levels were determined using a Mouse CX3CL1/Fractalkine DuoSet ELISA (R&D Systems, catalog number: DY472) following the manufacturer’s instructions [2]. Samples were diluted to a 1:2 in kit’s reagent diluent prior to plating. Each sample was plated in technical duplicates. Results were reported in picograms (pg) of fractalkine per milliliter (mL) of protein.

### Confocal microscopy

Tissues were imaged using a Zeiss LSM 710 microscope to generate a three-dimensional z-stack composite of retinal tissues. Composites of the image were generated using Imaris software v7.2 (Bitplane, RRID: SCR_007370). Three images per ¼ retina (1 image at the central retina nearest the optic nerve, 1 image in the middle of the leaflet, and 1 image in the outer leaflet) were analyzed per mouse. In addition, three images were obtained per optic nerve per mouse: 1 image proximal to the retina, 1 image in the middle of the optic nerve, and 1 image distal to the retina. Quantifications shown in graphical figures represent the average of the three images taken per mouse, spanning the three aforementioned regions of the retina averaging for Iba1^+^ cells/mm^3^, NeuN^+^ cells/mm^3^, percent immunoreactive area of CD31, fibrinogen, and optic nerve averaging percent immunoreactive area of TUJ1, SYP, and GFAP. To quantify Iba1^+^ microglial and NeuN^+^ neuronal cell body densities, cells were manually counted in 20 × images using the counter tool in Adobe Photoshop version 21.0.3. To quantify the percent immunoreactive area of CD31^+^ blood vessels and fibrinogen^+^, using ImageJ Fiji analysis software (NIH), raw confocal images (average of 3 images per retina per mouse) of the superficial plexus were converted to 32-bit grayscale, and then a global automatic threshold was applied to each image before computing the analysis. The percent area occupied by the automatic threshold was recorded as the percent immunoreactive area. Data were normalized by volume based on X, Y and Z coordinates (i.e., 425.1 µm × 425.1 µm × Z-stack thickness) to account for changes in confocal Z-stack thickness and images size (scale settings—distance in pixels: 1024; known distance: 412.5). To amplify regions of the optic nerve, images were taken at 63 × and the data for TUJ1^+^ axons, SYP^+^ synaptic vesicles, and GFAP^+^ astrocytes were normalized based on X, Y and Z coordinates (i.e., 134.95 µm × 134.95 µm × Z-stack thickness) to account for changes in confocal Z-stack thickness and images size (scale settings—distance in pixels: 1024; known distance: 130.9).

### Microglia morphology analysis

ImageJ analysis software (NIH) was used to determine the transformation index (TI) of 5 microglia per 20 × image using the equation: perimeter^2^/4π × area^2^ [3, 25].

### Flow cytometry

Peripheral blood was drawn by cheek puncture via submandibular vein and collected in 30 μL of 5000 U/mL heparin (Sigma-Aldrich, catalog number: H3393) in 1.5 mL Eppendorf tubes. Spleens were isolated and dissociated using 70 µm cell strainers. Blood and spleen erythrocytes were lysed under hypotonic conditions in water for 20 s followed by the addition of 10 × HBSS (supplemented with 10 mM HEPES) to bring the cells to a buffer. Cells were then centrifuged at 4 °C for 7 min at 2200 rpm and rinsed in 1 × HBSS. Brain and spinal cord tissues were isolated and homogenized in Gibco RPMI 1640 media (ThermoFisher Scientific, catalog number: 32404014), then resuspended in 10 mM HBSS/HEPES, followed by the isolation of mononuclear cells using Percoll gradients [26–28]. Cell suspensions were prepared in cell staining buffer (Biolegend, catalog number: 420201), following incubation with Fc block (BD Pharmingen, catalog number: 553142) for 15 min. Blood, spleen and CNS tissues were subjected to staining with antibody cocktails labeling peripheral leukocytes and CNS mononuclear cells with antibodies against CD11b, CD45, Ly6C, CD11c, (Additional file 1: Table S3) and adding Zombie aqua as a viability dye. Mononuclear cells from brain and spinal cord tissues were analyzed by flow cytometry using a mix of antibodies to identify activated microglia (CD45^Lo^CD11b^+^Ly6C^+^) and homeostatic microglia (CD45^Lo^CD11b^+^P2RY12^+^). For peripheral immune cells, macrophages were characterized as CD45^Hi^CD11b^+^Ly6C^+^, myeloid dendritic cells as CD45^Hi^CD11b^+^CD11c^+^, and conventional dendritic cells as CD45^Hi^CD11b^–^CD11c^+^. Splenic tissues were incubated with antibodies against CD11b, CD45, Ly6C, Ly6G, CD11c, MHC-II, CD3, CD4, CD8, CD44, CD25 (Additional file 1: Table S3), and Zombie aqua as a viability dye to identify granulocytes as CD45^Hi^CD11b^+^Ly6G^+^, inflammatory macrophages (with MHC-II antigen presentation) as CD45^Hi^CD11b^+^CD11c^–^Ly6C^+^, tissue resident macrophages as CD45^Hi^CD11b^+^CD11c^–^Ly6C^–^, conventional dendritic cells (with MHC-II antigen presentation) as CD45^Hi^CD11b^–^CD11c^+^, myeloid-derived dendritic cells (with MHC-II antigen presentation) as CD45^Hi^CD11b^+^CD11c^+^, CD4 T cells as CD45^Hi^CD11b^–^CD3^+^CD4^+^, activated CD4 T cells as CD45^Hi^CD11b^–^CD3^+^CD4^+^CD44^+^, CD4 T regulatory cells as CD45^Hi^CD11b^–^CD3^+^CD4^+^CD25^+^, CD8 T cells as CD45^Hi^CD11b^–^CD3^+^CD8^+^, activated CD8 T cells as CD45^Hi^CD11b^–^CD3^+^CD8^+^CD44^+^, and CD8 T regulatory cells as CD45^Hi^CD11b^–^CD3^+^CD8^+^CD25^+^. Cells were acquired in an LSR-II cytometer (BD Bioscience, RRID: SCR_002159) and analyzed using FlowJo software v9.2 (RRID: SCR_008520).

### RNA isolation and mRNA-seq analysis

RNA was isolated from enucleated retinas by the UT Health San Antonio Biospecimen and Translational Genomics Core Laboratory using the Qiagen RNeasy Kit (Qiagen, catalog number: 74104) following tissue homogenization using Zymo Research BashingBead lysis tubes (Zymo Research, catalog number: S6003-50). For mRNA sequencing, quality control (QC), stranded mRNA-seq library prep, and sequencing were performed by UT Health San Antonio Genome Sequencing Facility. The quality and assessment of Total RNAs, workflow, RNA-seq quantification process, and attainment of the average reads per sample was conducted as mentioned previously [3]. Raw reads were imported into CLC Genomics Workbench v21.0.5. Initially, adaptors were trimmed and remaining reads for each sample were mapped to the annotated mouse genome (GRCm39), followed by differential gene expression (DEG) analysis of diabetic mice with rAAV–mFKN or rAAV–sFKN against their diabetic FKN^KO^ controls, using the RNA-seq analysis tools within the CLC genomics software. DEG analysis was also performed on all diabetic groups at 4 wks and 10 wks. Genes were considered differentially expressed if the FDR *p* value was < 0.05. For data mining, DEGs were defined by Log_2_ fold values greater than or equal to 1 and less than or equal to − 1, and a cutoff FDR *p* value < 0.05.

### Visual acuity assessment

Individually housed mice were food deprived to 85% of their ad libitum body weight to increase motivation for the task. During the food deprivation period, mice were shaped to search for food reward and a cocoa puff (Cocoa Krispies, Kellogg’s) buried in a 30 mL cup, which limited their food intake prior to the commencement of the experiment [5]. Both the food pellet and the cocoa cereal crumb were hidden under cumin-scented, fine-grain woodchip bedding (Sani-Chip 146 IRR, LabSupply), to prevent the use of olfactory/reward associations. Mice were placed in the test arena after meeting their target weight, initiating their visual function assessment under visible light. The test arena was a rectangular plexi-glass apparatus (35.5 cm × 25.5 cm) with high and low complexity visual cues. A two-choice discrimination task featured one side of the box displaying four sinusoidal frequency spatial gradients, whereas the opposite side of the box showed two spatial gradients. Two plastic medicine cups that contained cumin-scented woodchip bedding were placed on opposite sides of the apparatus floor. The chocolate cereal crumb reward was always associated with the four-gradient wall. During the first three free-exploration pre-trials (Trials 1–3), the cocoa crumb was visibly shown on top of the cup next to the wall displaying four spatial gradients, which instructed mice to associate the spatial cues with the reward. The following four trials of every session were the experimental trials (Trials 4–7), during which the reward was hidden at a consistent depth beneath the top of the cup. To eliminate the use of extra-maze cues, including auditory and/or visual information, to find the reward, a white noise generator was used during training and testing and the arena was surrounded by a black curtain. Moreover, the test arena was rotated 90° clockwise from trial to trial to further limit the possibility that animals used extra-arena cues to find the reward. Finally, the task area was cleaned with 70% ethanol to eliminate animal scents after each trial. All trials were recorded with a LimeLight 4 video tracking system (Coulbourn Instruments, USA). Mice were left in the arena until a choice (correct or incorrect) was made. If no choice occurred in 3 min, animals were removed from the arena, and the trial was recorded as error. The experimental readouts tracked the location of the cup in which the animal fist dug after each trial and whether the animal ate the reward. In addition, control mice were assessed under infrared light to simulate conditions of blindness. To eliminate exposure to visible light cues, a red-light head lamp was used with handling mice in the dark during the inter-trial periods.

### Statistical analysis

All data are plotted as mean ± SD with scatter-dot plots to show the number and distribution of samples. Statistical analysis was performed in GraphPad Prism v10.0. Statistical tests performed included unpaired Student's *t* test with Welch’s correction when comparing two groups. Significant differences were denoted as *p value < 0.05, **p value < 0.01, ***p value < 0.001 and *****p value < 0.0001. All quantitative assessments were performed by a scientist blind to the experimental condition.

## Results

### rAAV effectively reconstituted fractalkine expression in FKN^KO^ retinal tissues

**Fig. 1:**
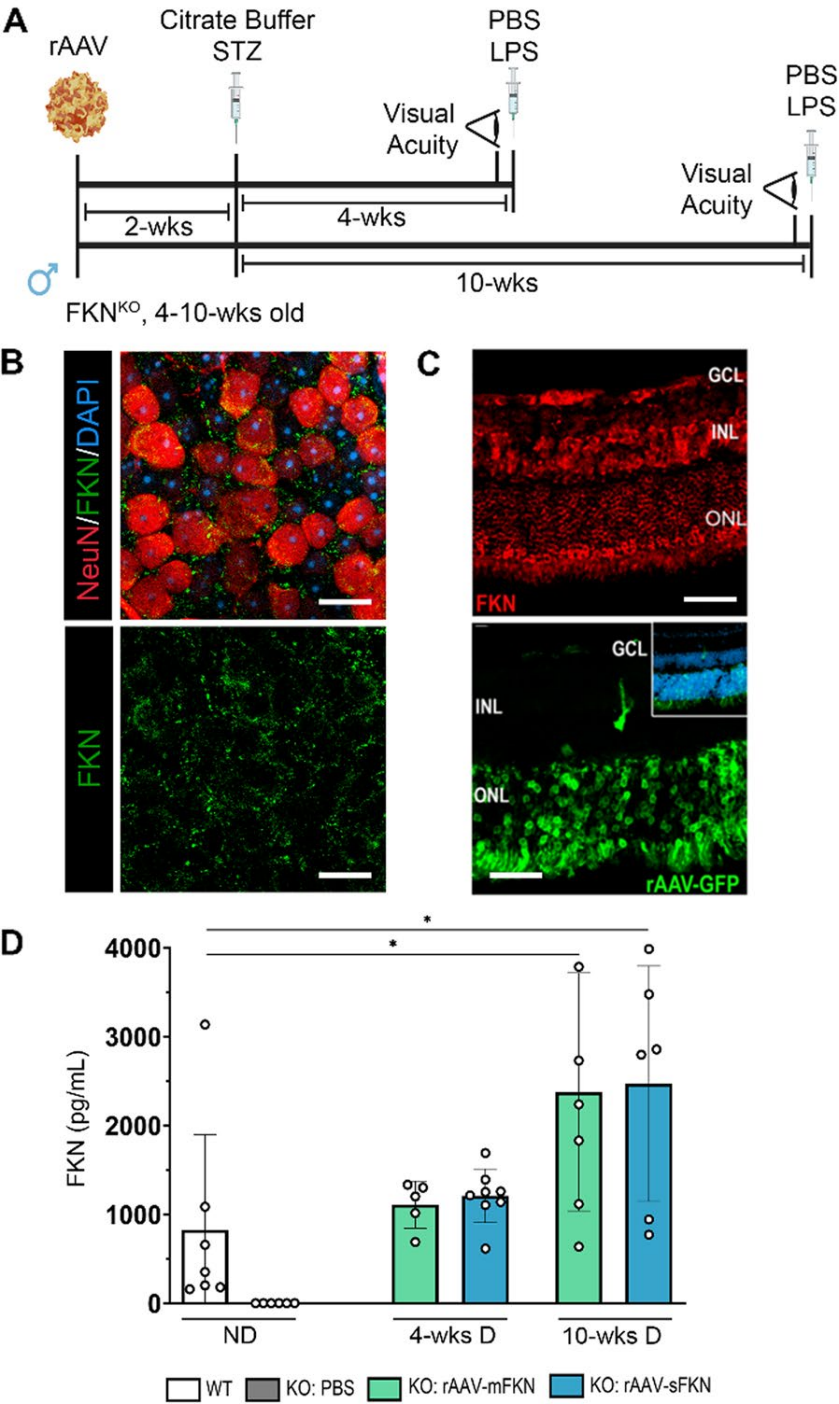
Assessing the effects of rAAV intra-vitreal delivery of mFKN or sFKN in the non-diabetic (ND) retina. **A** Experimental design to investigate the effects of intra-vitreal delivery of rAAV–mFKN or rAAV–sFKN to the retina. FKN^KO^ mice were given one intra-vitreal injection in the right eye of rAAV–mFKN or rAAV–sFKN (200 ng/mL). Two weeks after the intra-vitreal delivery of rAAV–mFKN or rAAV–sFKN, hyperglycemia was induced via intra-peritoneal streptozotocin (STZ) injections once daily for 5 days. To assess visual acuity, mice were shaped and placed in a test arena with high- and low-complexity visual cues, featuring a two-choice discrimination task. Two days before euthanasia, mice received intra-peritoneal lipopolysaccharide (LPS) injections once daily for 2 days, and tissues were collected at 4 weeks and 10 weeks of diabetes. **B** Confocal image of whole retina flat mount stained for cell nuclei (DAPI, blue), fractalkine (FKN, green), and NeuN (Red) (Scale bar; 20 µm). **C** Confocal image of cryosectioned retina stained for FKN (red, top panel) and endogenous rAAV–GFP virus delivered by intra-vitreal (green, bottom panel) (Scale bar; 50 µm). **D** Quantification of FKN (pg/mL) by ELISA in ND wild-type (WT) and PBS-treated FKN^KO^, 4-week diabetic and 10-week diabetic mice treated with rAAV–mFKN (green bars) or rAAV–sFKN (blue bars). Data shown as mean ± SD, *n* = 5–8 mice per group, where each dot represents an individual mouse across two experimental replicates. *p < 0.05 using Student’s *t* test, Welch’s correction

We delivered rAAV expressing mFKN or sFKN to FKN^KO^ retinas (Fig. 1A) at a concentration of 1 × 10^12^ vg/mL as previously described [24]. Six weeks after injection, retinal wholemount tissues from mice that received rAAV–mFKN were isolated, fixed, and stained with antibodies against FKN via immunofluorescence to visualize transduced cells showing FKN^+^ expression across neurons of the retina (Fig. 1B). FKN expression was evaluated in three layers surrounding the neuronal (NeuN^+^) cell bodies. Retinal layers were grouped by Z-stacks to show FKN expression in superficial (RGC layer to inner nuclear layer), intermediate (inner nuclear layer to outer nuclear layer), and deep (outer nuclear layer to retinal pigmented epithelium) layers (Additional file 1: Fig. S1A). FKN^+^ staining colocalized to NeuN^+^ cells confirming that the rAAV9 serotype used in all experiments was effective in targeting neurons and not glial or endothelial cells as previously reported [20]. Injection of rAAV harboring green fluorescent protein (rAAV–GFP) verified colocalization of rAAV expression to neurons within the ganglion cell, inner nuclear, and outer nuclear layers of the retina (Fig. 1C). Furthermore, FKN expression levels were validated at the protein level by ELISA (Fig. 1D). Retinal protein isolates from all groups were normalized to FKN^KO^ mice to eliminate assay unspecific binding. Protein isolates of mice that received rAAV–mFKN (1108 ± 264.7) and rAAV–sFKN (1207 ± 301.8) then subjected to sustained 4 weeks of diabetes (4-wk D) showed FKN levels comparable to the reference group, wild-type (WT) mice (823.7 ± 1073). FKN^KO^ mice that received rAAV–mFKN (2376 ± 1339) and rAAV–sFKN (2470 ± 1325) then subjected to sustained 10 weeks of diabetes (10-wk D) showed significantly higher FKN levels compared to WT mice (Student’s *t* test *p* < 0.05). These results confirmed that intra-vitreal delivery of FKN using rAAV lead to FKN expression levels comparable to WT mice, using in situ immunostaining in fixed tissues and ELISA assays in retinal protein extracts.

We also assessed the distribution of innate myeloid and lymphocyte populations in blood and spleens using flow cytometry to determine whether rAAV triggered systemic immune responses in diabetic animals (Additional File 1: Fig. S1B). We found that rAAV treatment in diabetic mice did not alter the frequencies of neutrophils (CD45^Hi^CD11b^+^SSC^Hi^), conventional (CD45^Hi^CD11b^–^CD11c^+^) or myeloid-derived (CD45^Hi^CD11b^+^CD11c^+^) dendritic cells (DCs) when compared to PBS-treated diabetic controls in the blood (Additional file 1: Fig. S1C–G). An increase in tissue-resident (CD45^Hi^CD11b^+^Ly6C^–^) and decrease in inflammatory (CD45^Hi^CD11b^+^Ly6C^+^) macrophages in diabetic mFKN-treated mice versus PBS-treated mice were detected (Additional file 1: Fig. S1D, E) (Student’s *t* test *p* < 0.05). An increase in blood neutrophils (Student’s *t* test *p* < 0.01), tissue-resident macrophages (Student’s *t* test *p* < 0.01), and a decrease in inflammatory macrophages (Student’s *t* test *p* < 0.01) and conventional DCs with rAAV–sFKN in non-diabetic (ND) mice. However, changes in splenic granulocytes (CD45^Hi^CD11b^+^Ly6G^+^), inflammatory (CD45^Hi^CD11b^+^CD11c^–^Ly6C^+^), tissue-resident (CD45^Hi^CD11b^+^CD11c^–^Ly6C^–^) macrophages (with respective MHC-II presentation), conventional (CD45^Hi^CD11b^–^CD11c^+^) or myeloid-derived (CD45^Hi^CD11b^+^CD11c^+^) DCs (with respective MHC-II presentation), CD4 T cells (CD45^Hi^CD11b^–^CD3^+^CD4^+^), CD4 activated T cells (CD45^Hi^CD11b^–^CD3^+^CD4^+^CD44^+^), CD4 T regulatory cells (CD45^Hi^CD11b^–^CD3^+^CD4^+^CD25^+^), CD8 T cells (CD45^Hi^CD11b^–^CD3^+^CD8^+^), CD8 activated T cells (CD45^Hi^CD11b^–^CD3^+^CD8^+^CD44^+^), and CD8 T regulatory cells (CD45^Hi^CD11b^–^CD3^+^CD8^+^CD25^+^) were not dependent on rAAV treatment, but rather the diabetic condition (Additional file 1: Fig. S2). These data suggest that intra-vitreal administration of rAAV–sFKN did not alter the systemic innate and adaptive cell profile during diabetes.

### sFKN prevented neuronal loss in the diabetic retina, and axonal damage and astrogliosis in optic nerves

**Fig. 2:**
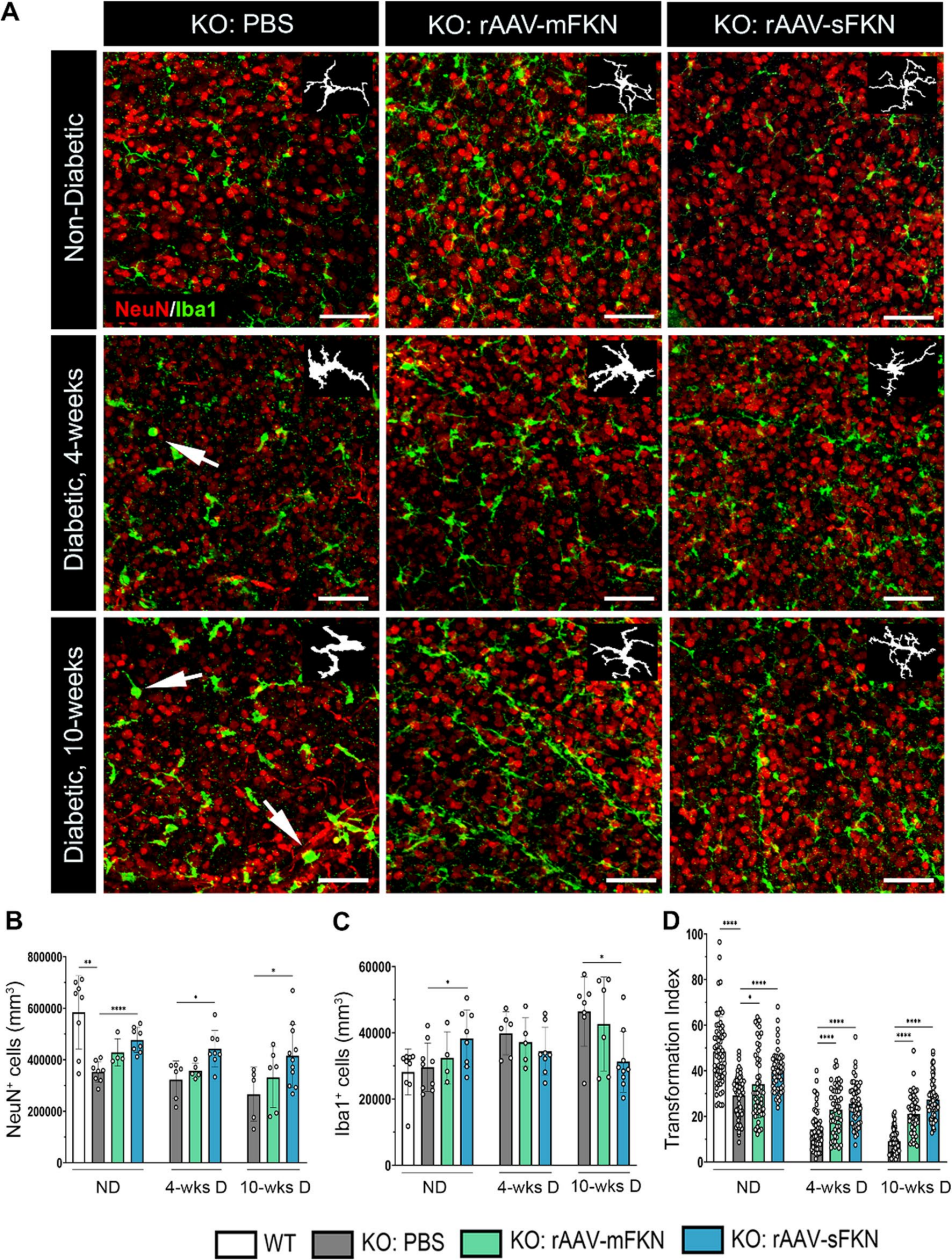
Intra-vitreal delivery of rAAV–sFKN prevents neuronal cell loss and microgliosis in the diabetic retina. **A** Confocal images of retinal tissues stained for NeuN (red) and Iba1 (green), with representation of cellular tracing for transformation index analysis (*Inset*) (Scale bar; 100 µm). **B**, **C** Quantification of retinal immunofluorescence analysis of NeuN^+^ cells/mm^3^ (**B**) and Iba1^+^ cells/mm^3^ (**C**). Data are shown as mean ± SD, *n* = 4–11 mice per group, where each dot represents an individual mouse across six experiments. **D** Transformation index quantification, where each dot represents an individual microglia cell *n* = 50–60 per group. Data shown as mean ± SD. *p < 0.05, **p < 0.01, and ****p < 0.0001 using Student’s *t* test, Welch’s correction

To assess the role of mFKN and sFKN on neurodegeneration during diabetes, retinal tissues were stained and imaged to analyze NeuN^+^ neuronal cell bodies (Fig. 2A, B). Analysis of NeuN^+^ cell densities revealed striking phenotypic differences in WT (583,879 ± 142,179) and FKN^KO^ (352,787 ± 40,647) in ND conditions, in which FKN^KO^ mice displayed a significant reduction in NeuN^+^ cells (Student’s *t* test *p* < 0.01) (Additional file 1: Fig. S3). Mice that received rAAV–sFKN (476,896 ± 48,750) showed a significant increase in NeuN^+^ cell densities compared to PBS-treated control mice (Student’s *t* test *p* < 0.0001). Interestingly, in diabetic groups intra-vitreal administration of rAAV–sFKN (442,448 ± 70,957), but not rAAV–mFKN showed a significant increase in NeuN^+^ cells compared to diabetic FKN^KO^ PBS-treated mice at 4-wk D (323,296 ± 71,592, Student’s *t* test *p* < 0.05) and 10-wk D (266,198 ± 105,326, Student’s *t* test *p* < 0.05) (Fig. 2B).

To further assess the neuroprotective effects of rAAV administration, optic nerve flat mounts were stained to visualize axonal integrity (TUJ1^+^), pre-synaptic integral membrane glycoprotein marker synaptophysin (SYP^+^), and astrocytes and Müller glia (GFAP^+^) at 10-wk D (Additional file 1: Fig. S4). Interestingly, ND FKN^KO^ PBS-treated (29.68 ± 6.59) optic nerves revealed decreased TUJ1^+^ immunoreactivity compared to WT tissues (40.03 ± 6.67, Student’s *t* test *p* < 0.05) (Additional file 1: Fig. S4) as an apparent cause due to loss of neurons in FKN^KO^ mice compared to WT mice (Additional file 1: Fig. S3). Under diabetic conditions, rAAV–sFKN (32.31 ± 2.87) groups showed an increased in TUJ1^+^ immunoreactivity compared to PBS-treated FKN^KO^ groups (Student’s *t* test *p* < 0.0001). Moreover, SYP^+^ immunoreactive area showed a significant increase with rAAV–mFKN (6.94 ± 1.39, Student’s *t* test *p* < 0.05) and rAAV–sFKN (9.69 ± 2.84, Student’s *t* test *p* < 0.01) compared to PBS-treated FKN^KO^ mice in ND groups (4.19 ± 2.05). Diabetic FKN^KO^ mice treated with rAAV–sFKN (7.25 ± 1.45), but not rAAV–mFKN (4.30 ± 0.99), showed a significant elevation in levels of SYP^+^ immunoreactivity compared to diabetic PBS-treated FKN^KO^ mice (3.90 ± 1.18, Student’s *t* test *p* < 0.0001) (Additional file 1: Fig. S4). We observed an increase in GFAP^+^ immunoreactive area in ND PBS-treated FKN^KO^ mice (28.50 ± 3.67) compared to their WT controls (18.94 ± 1.27, Student’s *t* test *p* < 0.001) (Additional file 1: Fig. S4). However, rAAV–sFKN (20.98 ± 2.03) administration significantly decreased GFAP^+^ immunoreactivity in ND groups, compared to ND FKN^KO^ mice (28.50 ± 3.67, Student’s *t* test *p* < 0.01). Diabetes significantly elevated GFAP^+^ immunoreactivity in FKN^KO^ mice (33.82 ± 4.54), which was decreased with rAAV–sFKN (23.51 ± 3.02, Student’s *t* test *p* < 0.01) but not rAAV–mFKN (30.20 ± 3.36). This data suggests that rAAV–sFKN therapy is consistently neuroprotective in the FKN^KO^ retina under ND and diabetic conditions. Furthermore, rAAV–sFKN can support the integrity of the optic nerve by preventing neuronal damage and transmitter release, and astrogliosis during DR pathological conditions.

### sFKN consistently reduced microgliosis and microglial morphological activation

To evaluate the role of mFKN and sFKN on microglia cell densities and activation during diabetes, retinal tissues were stained and imaged to analyze Iba1^+^ microglia cell (Fig. 2A) in different stages of morphological activation, where a high transformation index (TI) value represents homeostatic, ramified microglia, and a low TI represents ameboid, activated microglia. In ND groups, rAAV–sFKN (38,862 ± 8,622) significantly increased microglia cell counts compared to PBS-treated FKN^KO^ mice (29,637 ± 7,311, Student’s *t* test *p* < 0.05) (Additional file 1: Fig. S3). Microglia cell densities in control PBS-treated FKN^KO^ mice, rAAV–mFKN and rAAV–sFKN groups showed no significant changes at 4-wk D. At 10-wk D, rAAV–sFKN (31,301 ± 9,193), but not rAAV–mFKN (42,687 ± 14,160), significantly reduced Iba1^+^ cells in the retina compared to PBS-treated FKN^KO^ mice (46,446 ± 10,468, Student’s *t* test *p* < 0.05) (Fig. 2C). This data suggests that sFKN mitigates the effects of microgliosis during diabetes. Further investigating the effects of mFKN and sFKN on microglial activation, morphometric analysis of retinal microglia were conducted using the TI of Iba1^+^ cells (Fig. 2A, *Inset*). ND PBS-treated FKN^KO^ mice (29.24 ± 9.29) showed a significant decrease in the microglial TI compared to WT mice (48.45 ± 15.11, Student’s *t* test *p* < 0.0001). Moreover, microglial TI significantly increased with the administration of rAAV–mFKN (34.23 ± 14.83, Student’s *t* test *p* < 0.05) and rAAV–sFKN (39.72 ± 9.31, Student’s *t* test *p* < 0.0001) in ND mice compared to ND PBS-treated FKN^KO^ mice. Expression of mFKN (23.02 ± 10.63) or sFKN (25.63 ± 9.52) induced a significant increase in microglial TI cells at 4-wk D compared to PBS-treated FKN^KO^ mice (14.30 ± 8.80, Student’s *t* test *p* < 0.0001) (Fig. 2D). Likewise, rAAV delivery of mFKN (21.06 ± 8.58) and sFKN (27.39 ± 8.77) compared to PBS-treated FKN^KO^ mice (9.07 ± 5.57, Student’s *t* test *p* < 0.0001) showed a significant increase in microglial TI cells at 10-wk D. (Fig. 2D). These results show that with the administration of rAAV–sFKN or rAAV–mFKN, microglia are phenotypically less ameboid at both 4-wk D and 10-wk D.

To validate microglia activation in overall CNS tissues, flow cytometry on brain and spinal cord was used to distinguish homeostatic (CD11b^+^CD45^Lo^P2RY12^+^Ly6C^–^) versus reactive (CD11b^+^CD45^Lo^P2RY12^+^Ly6C^+^) microglia (Additional file 1: Fig. S5). Treatment with rAAV–mFKN (98.95 ± 0.56, Student’s *t* test *p* < 0.01) and rAAV–sFKN (98.87 ± 0.59, Student’s *t* test *p* < 0.05) during diabetes demonstrated a significant increase in homeostatic microglia compared to PBS-treated FKN^KO^ mice (96.93 ± 0.56) (Additional file 1: Fig. S5). These results are consistent with the microglia phenotype observed in immunofluorescent analysis of Iba1^+^ and TI analysis, suggesting that rAAV can spread, leading to FKN overexpression in the CNS to mediate microglia hyperactivity.

### sFKN ameliorated vascular damage and fibrin(ogen) deposition in the diabetic retina

**Fig. 3:**
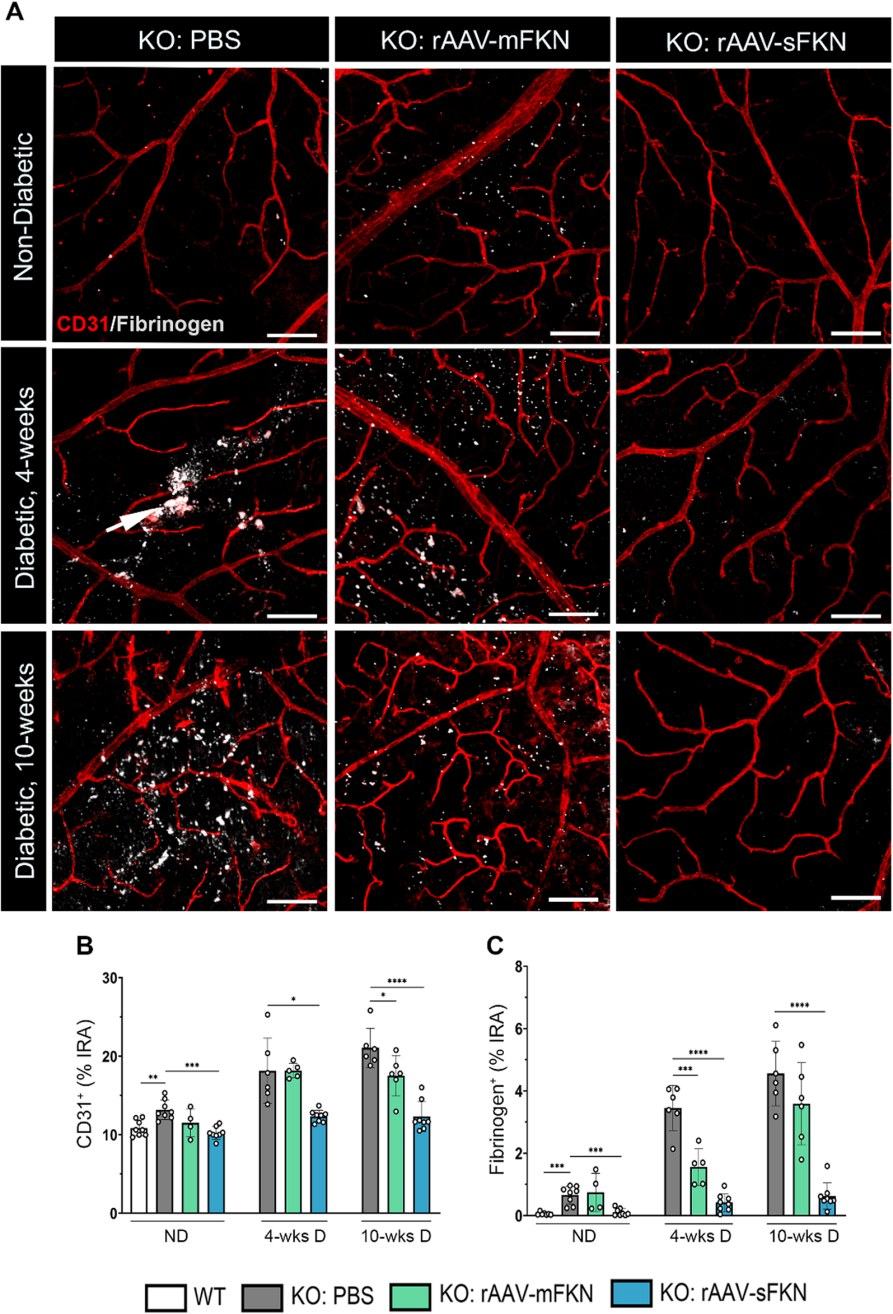
Intra-vitreal administration of rAAV–sFKN reduces vascular pathology and fibrin(ogen) deposition in the diabetic retina. **A** Confocal images of retinal tissues stained for CD31 (red) and fibrinogen (white) (Scale bar; 100 µm). **B**, **C** Quantification of retinal immunofluorescence analysis of CD31^+^ percent immunoreactive area (% IRA) (**B**) and fibrinogen^+^ immunoreactive area (% IRA) (**C**). Data shown as mean ± SD, *n* = 4–8 mice per group, where each dot represents an individual mouse across six experiments. *p < 0.05, **p < 0.01, ***p < 0.001 and ****p < 0.0001 using Student’s *t* test, Welch’s correction

We sought to determine the role of mFKN and sFKN in the context of vascular pathology and as a readout of vascular rupture and serum protein leakage. Utilizing high-resolution confocal, we compared the CD31^+^ and fibrinogen^+^ immunoreactive area and identified ruptured blood vessels containing fibrinogen^+^ deposits outside of the vasculature (Fig. 3A). ND PBS-treated FKN^KO^ mice (13.17 ± 1.24) showed significantly higher CD31^+^ endothelial cell percent immunoreactive area compared to WT mice (10.85 ± 1.01, Student’s *t* test *p* < 0.01) (Additional file 1: Fig. S3). Intra-vitreal injection of rAAV–sFKN (10.22 ± 0.78), but not rAAV–mFKN (11.50 ± 1.77) decreased CD31^+^ immunoreactive area compared to PBS-treated FKN^KO^ mice (Student’s *t* test *p* < 0.001). Delivery of rAAV–sFKN (12.34 ± 0.76) but not rAAV–mFKN (18.15 ± 0.93) significantly decreased vascular pathology compared to PBS-treated FKN^KO^ mice at 4-wk D (18.12 ± 4.16, Student’s *t* test *p* < 0.05). Furthermore, mFKN (17.51 ± 2.58, Student’s *t* test *p* < 0.05) and sFKN (12.31 ± 1.92, Student’s *t* test *p* < 0.0001) showed decreased immunoreactivity of CD31^+^ compared to FKN^KO^ mice at 10-wk D (21.06 ± 2.50) (Fig. 3B). Overall, these data suggest that treatment with rAAV–sFKN dependably reduces neovascularization at both diabetic timepoints.

Vascular damage in diabetic retinas was assessed by quantifying fibrin(ogen) deposition in the retinal parenchyma around the injured vasculature. Results showed substantial extravasation of fibrin(ogen) in ND PBS-treated FKN^KO^ mice (0.66 ± 0.28) compared to WT mice (0.05 ± 0.04, Student’s *t* test *p* < 0.001) (Additional file 1: Fig. S3). In ND mice, fibrin(ogen) deposition was ameliorated by rAAV–sFKN (0.10 ± 0.11) but not rAAV–mFKN (0.74 ± 0.61), when compared to PBS-treated FKN^KO^ mice (Student’s *t* test *p* < 0.001). Fibrin(ogen) aggregates in PBS-treated FKN^KO^ mice (3.45 ± 0.72) significantly decreased with rAAV–mFKN (1.55 ± 0.58, Student’s *t* test *p* < 0.001) and rAAV–sFKN (0.42 ± 0.28, Student’s *t* test *p* < 0.0001) at 4-wk D. PBS-treated FKN^KO^ mice (4.55 ± 1.03) at 10-wk D showed profound fibrin(ogen) deposition into the retinal parenchyma which was significantly reduced with rAAV–sFKN (0.62 ± 0.42, Student’s *t* test *p* < 0.0001), but not in rAAV–mFKN treated groups (3.58 ± 1.31) (Fig. 3C). These results indicated that sFKN is consistently vasculo-protective, reducing vascular pathology and fibrin(ogen) deposition, at both diabetic timepoints.

### Gene expression analyses revealed sFKN regulates microglia-mediated inflammation and induces positive regulation of apoptosis

**Fig. 4:**
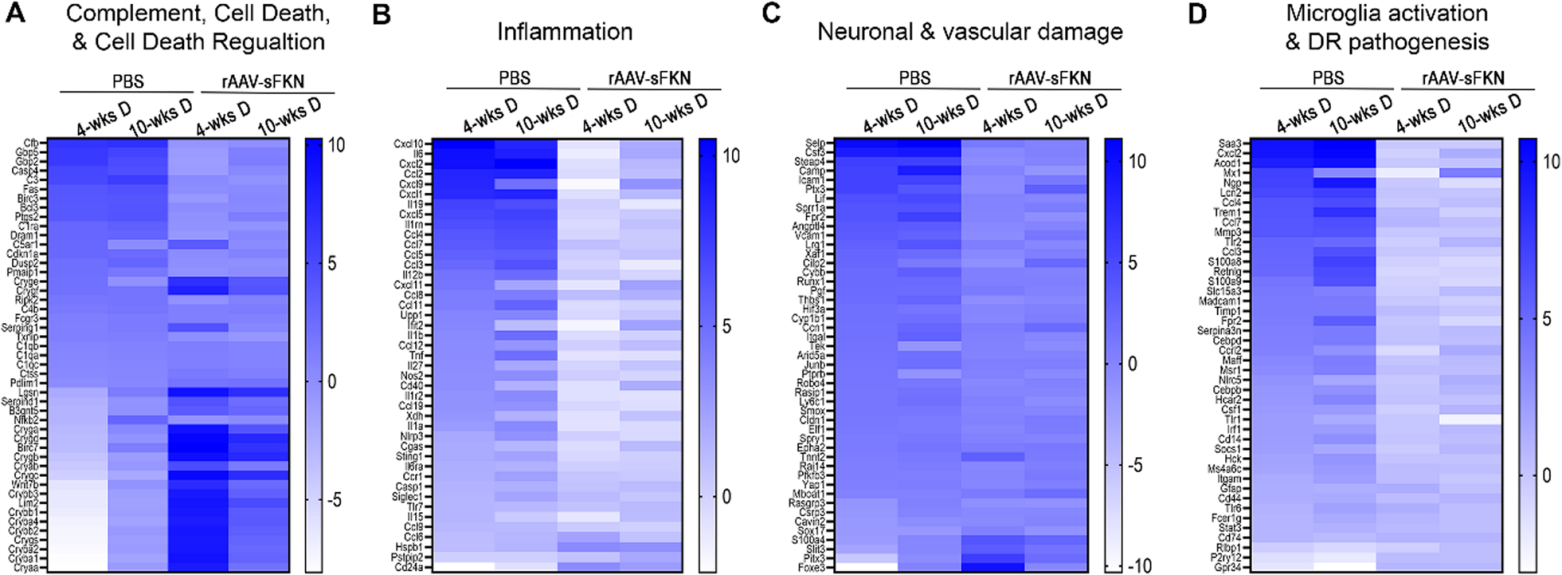
rAAV–sFKN alleviates microglia-mediated inflammation in the diabetic retinal transcriptome. **A–D** Heat map gene expression of all up and down regulated DEGs associated with the complement pathway, cell death and cell death regulation (**A**), inflammatory cytokines and chemokines (**B**), vascular and neuronal damage (**C**) and microglia activation and DR pathogenesis (**D**). *n* = 4–5 mice per treatment group across two experiments. All RNA samples were processed at the same time. All heat maps compared PBS-treated 4-wk D and 10-wk D mice to PBS-treated ND controls, and PBS-treated 4-wk D and 10-wk D mice to rAAV–sFKN-treated mice

To determine the effect of rAAV on neuroinflammatory responses, we performed mRNA-seq on whole retinas (Fig. 4 and Additional file 1: Figs. S6–S8). Up and down regulated genes are presented in heat maps to present pathways of complement, cell death, and cell death regulation (Fig. 4A), inflammation (Fig. 4B), neuronal and vascular damage (Fig. 4C), and microglia activation and DR pathogenesis (Fig. 4D). In PBS-treated mice at 4-wk D (compared to their ND PBS-treated controls) we identified 1,571 upregulated differentially expressed genes (DEGs) and 833 downregulated DEGs (Additional file 1: Fig. S7A-B). PBS-treated 10-wk D mice showed 1,677 upregulated DEGs and 6,935 DEGs downregulated DEGs relative to the ND PBS-treated controls (Additional file 1: Fig. S7C-D). Mice at 4-wk D had 102 significant differentially expressed genes (DEGs, false discovery rate (FDR) < 0.05, log2 (fold change) ≥ 1.0 or ≤  − 1.0) and 10-wk D mice had 75 significant DEGs compared to their ND PBS-treated groups. Compared to baseline levels of ND PBS-treated mice, analysis of PBS-treated 4-wk D retinas revealed significant increase in DEGs associated with decreased regulation of cell death (*Cryba1*, *Cryba2*, *Crygs*, *Crybb2*, *Cryba4*, and *Crybb1*) and increase in complement activation (*Fas*, *C3*, *C1ra*, and *Cfb*) (Additional file 1: Fig. S6A), increase inflammation (*Il15*, *Il1a*, *Tnf*, *Il1b*, *Il12b*, and *Il6*) (Additional file: Fig. S6B), increased vascular and neuronal damage (*Ly6c1*, *Cyb1b1*, *Cybb*, *Vcam1*, *Ptx3*, *Icam1*, and *Csf3*) (Additional file 1: Fig. S6C), and increase expression of microglia activation and DR pathogenesis (*Csf1*, *Cebpb*, *Ccrl2*, *Serpina3n*, *Frp1*, *Madcam1*, *Acod1*, and *Saa3*) (Additional file 1: Fig. S6D). Analysis of PBS-treated 10-wk D retinas revealed significant increase in DEGs associated with decreased regulation of cell death and complement activation (*C1ra*, *C5ar1*, *Bcl3*, *Fas*, *C3*, and *Cfb*) (Additional file 1: Fig. S6E), inflammation (*Nos2*, *Tnf*, *Il12b*, *Ccl5*, *Ccl7*, *Ilrn*, and *Il6*) (Additional file 1: Fig. S6F), increased vascular and neuronal damage (*Ly6c1*, *Ccn1*, *Cdkn1a*, *Vcam1*, *Ctla2a*, *Icam1*, *Steap4*, and *Csf3*) (Additional file 1: Fig. S6G), and increase microglia activation and DR pathogenesis (*Csf1*, *Cebpb*, *Msr1*, *Serpina3n*, *Fpr2*, *Lcn2*, *Acod1*, and *Saa3*) (Additional file 1: Fig. S6H).

We identified 637 upregulated DEGs and 610 downregulated DEGs in mice that were 4-wk D with rAAV–sFKN (Additional file 1: Fig. S7I-J) and 410 upregulated DEGs and 155 DEGs downregulated DEGs in mice that were 10-wk D with rAAV–sFKN relative to their respective diabetic timepoints with PBS (Additional file 1: Fig. S7K-L). Of those genes, 4-wk D mice administered with rAAV–sFKN had 26 significant DEGs, and 10-wk D mice treated with rAAV–sFKN had 5 significant DEGs compared to their diabetic PBS-treated groups. Genes associated with regulation of cell death (*Cryab*, *Cryge*, *Crygf*, *Cryba4*, *Cryaa*, *Crybb1*, *Crygs*, *Cryba2*, *Crybb2*, *Cryba1*, *Cryga*, *Crygb*, *Crygc*, and *Crygd*) (Additional file 1: Fig. S6I), inflammation (*Hspb1* and *Cd24a*), (Additional file 1: Fig. S6J), and vascular and neuronal damage (*Tnnt2*, *Slit3*, and *S100a4*) (Additional file 1: Fig. S6K) were significantly upregulated in rAAV–sFKN treatment mice relative to PBS-treated 4-wk D mice. Interestingly, genes associated regulation of cell death (*Crygc*, *Crygb*, and *Crygd*) (Additional file 1: Fig. S6L), were significantly upregulated in rAAV–sFKN treatment mice relative to PBS-treated 10-wk D mice. Altogether, these findings corroborated that rAAV–sFKN therapy is neuroprotective by attenuating microglia-mediated activation, reducing inflammation, which suggests a neuroprotective mechanism by inducing anti-apoptotic genes.

### Diabetic FKN^KO^ mice demonstrated enhanced vision loss that is rescued by sFKN

**Fig. 5:**
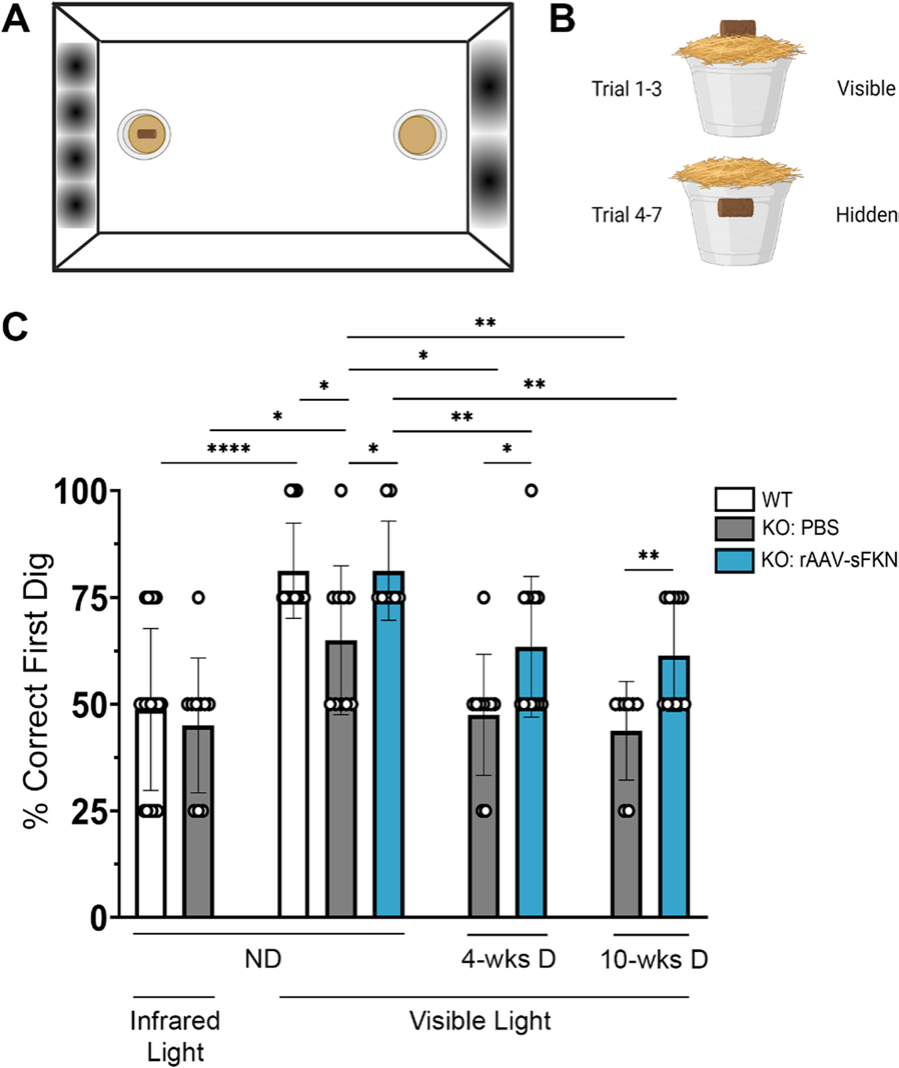
rAAV–sFKN administration prevents loss of visual acuity in diabetic FKN^KO^ mice. **A**, **B** Schematic layout of the visual acuity task displaying distinct sinusoidal spatial frequencies and location of the food reward during each trial in the two-choice discrimination task arena. **C** Quantification of correct first dig location (%). Data shown as mean ± SD, *n* = 8–20 mice per group, where each data point represents an individual mouse across three experimental replicates. *P < 0.05, **p < 0.01, and ****p < 0.0001 using Student’s *t* test, Welch’s correction

Next, we asked if administration of rAAV–sFKN influenced visual function. A visual acuity assessment, featuring a two-choice discrimination task (Fig. 5A, B) was utilized to determine if mice could distinguish between two distinct visual cues [5, 29]. ND WT and FKN^KO^ PBS-treated animals were tested under visible and infrared light to establish a baseline of visual acuity readouts. Infrared light was used to mimic conditions of visual impairment. ND WT (48.75% ± 18.95%) and FKN^KO^ (45.00% ± 15.81%) mice tested under infrared light, identified the reward less than 50% of the trials (Fig. 5C) performing at chance level. This indicated the animals’ inability to distinguish between spatial cues to locate the reward. Under visible light conditions, the same groups of mice performed the task successfully. The ratio of correct digs was lower in FKN^KO^ mice (65.00% ± 17.48%) compared to WT mice (81.25% ± 11.11%, Student’s *t* test *p* < 0.05). After establishment of baseline readout in these mice, we assessed visual acuity in ND and diabetic PBS-treated or rAAV–sFKN-treated mice. ND FKN^KO^ mice with rAAV–sFKN (81.25% ± 11.57%) showed similar percentage as the WT group. FKN^KO^ mice at 4-wk D (47.50% ± 14.19%) and 10-wk D (43.75% ± 11.57%) showed worse visual acuity, comparable to PBS-treated FKN^KO^ mice in infrared light. Mice with rAAV–sFKN showed better performance than their PBS-treated FKN^KO^ counterparts at 4-wk D (63.46% ± 16.51%, Student’s *t* test *p* < 0.05) and at 10-wk D (61.36% ± 13.06%, Student’s *t* test *p* < 0.01) (Fig. 5C). These data suggest that treatment with rAAV–sFKN improves visual acuity, further supporting previous findings of reduced vascular and neuronal damage via regulation of inflammation.

## Discussion

The present study offers compelling evidence supporting the role of sFKN signaling in the amelioration of retinal pathology. Through a viral approach, we examined the impact of mFKN and sFKN on microglia-mediated retinal inflammation during the onset and early stages of diabetes. Significantly, our findings demonstrate that sFKN confers neural and vascular protection leading to reduced inflammation and improved visual function. These results imply that targeting microglia pro-inflammatory responses could serve as a promising therapeutic strategy for managing vascular damage and neurodegeneration in diabetic retinas. The significant therapeutic potential of unraveling molecular cascades for the targeted delivery of viral treatments to alleviate DR emphasizes the importance of these findings.

The study described here complements our previous work, showing that disruption of CX3CR1/FKN signaling correlated with altered microglia morphology, perivascular clustering, and proliferation, resulting in augmented loss of retinal neurons [2–4, 30, 31]. The introduction of the hCX3CR1^I249/M280^ variant into the mouse genome and the utilization of CX3CR1^KO^ and FKN^KO^ mouse lines exposed under-appreciated roles of CX3CR1/FKN signaling in DR via the regulation of inflammatory processes [4]. Moreover, post-mortem retinas of diabetic patients have shown signs of inflammation, increased gliosis and vascular aberrations [5]. Together, these models showed: (1) Neuronal loss associated with increased microglia activation in diabetic CX3CR1^KO^ and hCX3CR1^I249/M280^ mice; (2) Increased vascular injury, defined by microglial clusters colocalizing to fibrin(ogen) deposition in CX3CR1^KO^ and hCX3CR1^I249/M280^ mouse retinas; (3) The prominent expression of IL-1β in CX3CR1^KO^ diabetic retinas; (4) Amelioration of neuronal and vascular damage by microglia depletion in the CX3CR1^WT^ diabetic retina; and (5) Significant microglial activation and fibrin(ogen) immunoreactivity in human diabetic retinas, highlighting signs of inflammation, neuroglial dysfunction, and vascular alterations [3–5]. The present study provides additional evidence that signaling mediated by sFKN ameliorates retinal pathology.

Most accepted ophthalmic procedures for treating DR involve intra-vitreal delivery of anti-inflammatory steroids, VEGF and IL-1β inhibitors, and panretinal laser photocoagulation therapies. These strategies are mainly employed at chronic stages of disease [10, 20, 32–37]. The lack of mechanistic knowledge on disease initiation hinders attempts to treat early DR. The DR field is still searching for interventions to reverse tissue damage and visual function. Gene therapy using rAAV is used to normalize, restore, and express genetic sequences to induce therapeutic results in ophthalmic diseases. Luxturna™, an FDA-approved therapy for treating congenital amaurosis, is a vector-based gene therapy that reconstitutes gene RPE65 in retinal cells with RPE65 mutations [38]. Our study was conceived based on the previous novel outcome showing that treatment of recombinant sFKN in the retina decreased microglia activation, perivascular clustering, and fibrin(ogen) deposition in mice lacking FKN and in a model of DR [4]. Here, we sought to disentangle the contribution of mFKN and sFKN using an rAAV platform, which has enormous therapeutic potential to characterize the role of these signaling pathways as regulators of inflammation and treatments for vision loss in DR.

Diabetes-driven retinal neurodegeneration is involved in DR [39, 40]. To test the hypothesis that neuronal-derived FKN can ameliorate neurodegeneration in a mouse model of STZ-induced diabetes, vectors expressing sFKN or mFKN were delivered intra-vitreally in the retina. rAAV–GFP harboring green fluorescent protein allowed us to confirm the colocalization of these vectors in neuronal cells (Fig. 1C). By immunostaining and ELISA, we validated that rAAVs yielded higher protein expression levels in comparison with those of WT animals (Fig. 1D). Neuronal densities were higher in rAAV–sFKN treated ND and diabetic mice (Fig. 2B). The observation of reduced neuronal densities in the retinas of ND FKN^KO^ mice was surprising but aligned with published data. FKN^KO^ mice showed significant deficits in hippocampal neurogenesis [24]. Similarly, the reduced density of TUJ1^+^ axons and SYP^+^ presynaptic vesicle staining in ND PBS-treated FKN^KO^ optic nerves highlights the functional relevance of this chemokine in neuronal homeostasis. In 10-wk D animals, administration of rAAV–sFKN correlated with increased neuronal densities in the retina, and optic nerve TUJ1^+^ and SYP^+^ immunoreactivity were also elevated to levels comparable to ND WT mice.

Retinal inflammation plays a significant role in DR. A distinct function of FKN within the CNS is to reduce microglial activation and associated pro-inflammatory responses. Interestingly, rAAV–sFKN treatment in ND groups increased microglia densities compared to PBS-treated controls (Fig. 2C). This is likely due to the local innate immune response of resident microglia to the rAAV vectors. The observation that the densities of blood-derived infiltrating cells were similar in the CNS of the ND PBS-treated and rAAV–sFKN-treated groups also supports the rationale that rAAVs do not induce exaggerated inflammation. To further confirm that rAAVs do not alter immune responses, peripheral immune cell populations were assessed by flow cytometry in blood samples. The data showed increased neutrophil and tissue-resident macrophage densities in ND mice treated with rAAV–sFKN (Additional file 1: Fig. S1C, D). However, no statistically significant differences were observed in the densities of inflammatory macrophages or dendritic cells in the diabetic groups treated with rAAV–sFKN compared to PBS-treated controls. Similarly, densities of splenic innate immune cells (neutrophils, macrophages, and dendritic cells) and T cells were not altered by the rAAV–sFKN treatment (Additional file 1: Fig. S2). Overall, rAAV–sFKN treatment did not significantly alter the patterns of peripheral innate and adaptive immune cells in our model. Retinal Iba1^+^ total microglial densities increased due to diabetes (10 wks), and treatment with rAAV–sFKN ameliorated microgliosis in diabetic groups. Surveillant microglia continuously extend and retract their fine processes in the healthy CNS [41, 42]. This movement of microglial processes is assumed to play roles in monitoring extracellular spaces for pathogens or damaged cells. The transformation of microglia from highly branched cells to ameboid, is captured by morphometric studies including the transformation index to detect morphological changes known to correlate with specific inflammatory phenotypes [43]. Furthermore, to compare activated and homeostatic microglia, flow cytometry showed that the Ly6C^+^ microglia population was significantly reduced in rAAV–sFKN groups, whereas the homeostatic P2RY12^+^ microglia populations were increased (Additional file 1: Fig. S5). Moreover, rAAV–sFKN administration restored microglial morphological activation in diabetic mice to levels comparable to ND controls.

Neuroinflammatory processes can be treated using rAAV therapies to regulate microglial environments. For instance, in mouse models of Alzheimer's disease, overexpression of sFKN using rAAV in Tg4510 mice (but not in APP/PS1 mice) led to reduced tau phosphorylation and deposition, decreased neuronal loss and brain atrophy, and cognitive improvements [34, 35]. Interestingly, rats treated with sFKN showed less neurodegeneration compared to animals treated with mFKN or untreated rats, irrespective of MHC-II expression, suggesting that sFKN likely shifts the microglial environment toward a neuroprotective state rather than solely reducing antigen expression [35]. Furthermore, FKN^KO^ mice displayed reduced synaptic plasticity, decreased neurogenesis, defective myelination, and profound cognitive impairments in memory and spatial tasks [24]. Notably, the delivery of sFKN, but not mFKN, improved hippocampus-dependent long-term memory [24]. The positive effects of FKN have also been observed in motor and visual systems. Administration of rAAV–sFKN in the substantia nigra protected dopaminergic neurons and improved motor function by reducing microglia activation in Parkinson's disease models. In addition, viral expression of sFKN, but not full-length FKN, rescued cone loss in a mouse model of retinitis pigmentosa [44]. In summary, previous studies have demonstrated that both mFKN and sFKN play pivotal roles in cognition, neurodegeneration, and sensory processing. Our research extends these findings by distinguishing the relative contributions of mFKN and sFKN and suggesting a potential therapeutic role for sFKN in treating retinal damage and preventing microglia-mediated inflammation and neurodegeneration.

Our data also demonstrate that microglia activation correlates with the degree of neuronal loss and vascular leakage. Results from this study are consistent with former discoveries in the context of diabetes using the Ins2^Akita^ model, STZ model, and microglia depletion model showing dysregulated microglial responses in absence of CX3CR1/FKN signaling [2–5]. Treatment of mFKN or sFKN altered microglia morphology, indicating the adoption of homeostatic phenotype compared to PBS-treated FKN^KO^ mice. Complementary to these findings, we assessed the association between vascular abnormalities and fibrin(ogen) deposition in retinal flat mounts (Fig. 3). Our research suggests sFKN treatment, diminishing the extravasation of fibrin(ogen) and vascular pathology by attenuating microglia dysregulation in the retinal microenvironment, resolving vascular damage. We speculate that sFKN therapy can enhance CX3CR1/FKN singling to promote vasoconstriction in the diabetic retina [31].

In diabetic patients, uncontrolled systemic inflammation propagates microglia activation [5, 45]. We have characterized microglia phenotypes in relation to their activation by identifying their ameboid appearance with truncated, retracted cellular processes (Fig. 2D). Treatment with rAAV–sFKN resulted in ramified microglia with long cellular processes in diabetic mice, resembling ND mice. We have shown that early signs of vascular and neuronal damage occur as early as 4-wk D (Figs. 2 and ) due to microglia release of IL-1β, TNF-α, and NOS2. The relationship between cell death and upregulation of crystallin genes in rAAV–sFKN groups illuminates potential pathways to explain how sFKN can ameliorate inflammation early in DR (Additional file 1: Fig. S6I, L). Of interest was the significant upregulation of complement genes, particularly C3 and C1q [3], and downregulation in rAAV–sFKN treated mice. Microglia are significant contributors to C1q and C3 production in response to inflammation and injury [46, 47]. Astrocytes and neurons can also express C1q [48]. However, little is known about the potential crosstalk between cell types mediated by complement molecules, particularly since the microglial-mediated release of pro-inflammatory cytokines such as IL-1β and TNF-α can stimulate complement molecules. Conversely, anti-inflammatory cytokines, such as IL-4 and IL-10, can suppress complement expression [46, 49]. Due to the connection between complement molecules, fibrin(ogen), and blood–retinal barrier integrity, our data supports the rationale that sFKN sustains a population of microglia with a homeostatic gene expression profile that supports retinal function.

**Fig. 6:**
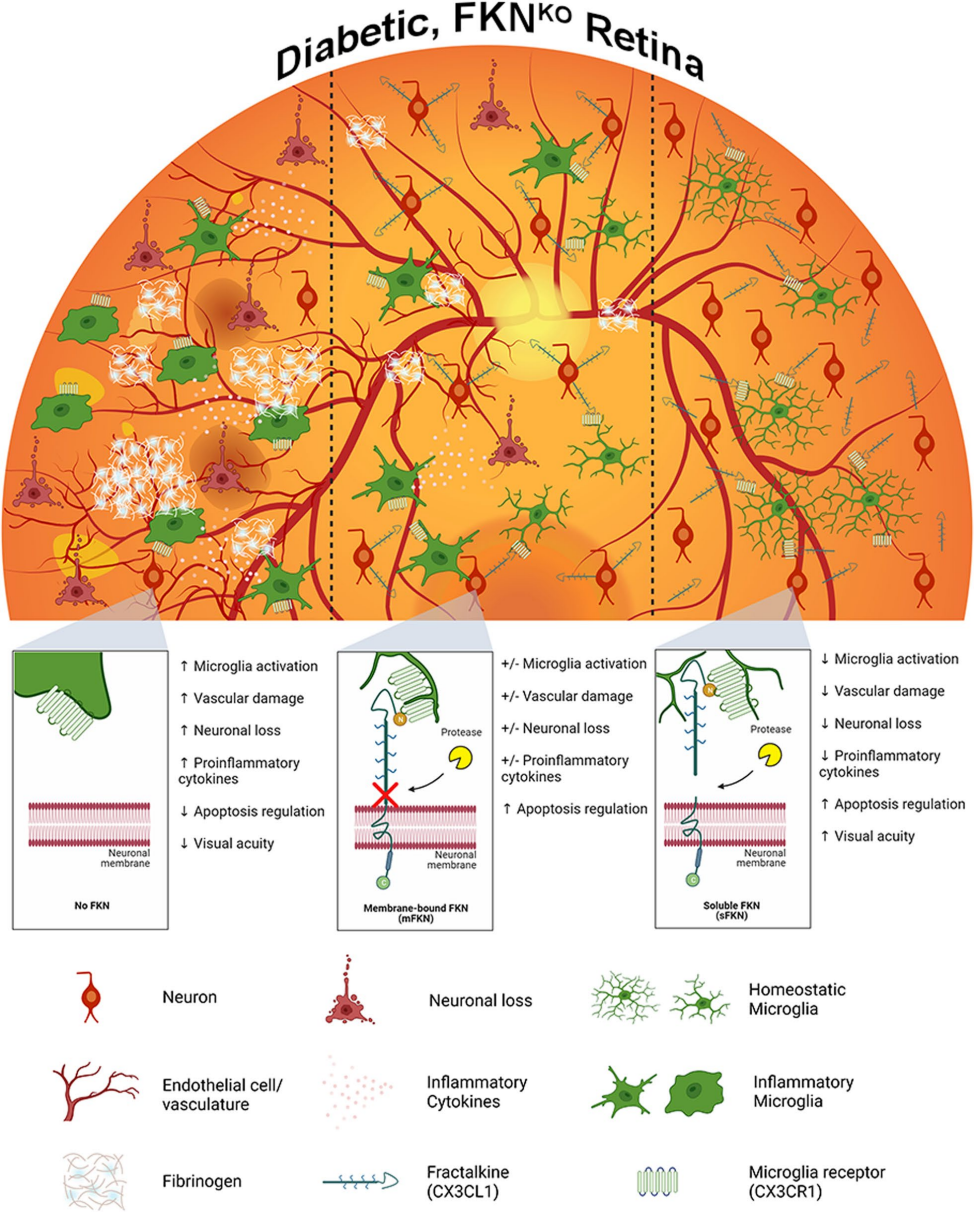
Schematic summary. Prolonged hyperglycemia using the two-hit inflammatory model results in increased pro-inflammatory microglia, resulting in neuronal cell loss, vascular pathology, and fibrinogen deposition in PBS-treated FKN^KO^ mice (left panel). In addition, diabetic rAAV–mFKN treated FKN^KO^ mice (no FKN cleavage occurring) shows more or less neuroprotection, microglia activation, and vascular damage (middle panel). Intra-vitreal administration of rAAV–sFKN alleviates microglia activation, preventing vascular damage, fibrin(ogen) deposition, and neuronal loss in the diabetic FKN^KO^ retina (right panel)

FKN isoforms offer various avenues for modulating the glial profile. Overall, sFKN emerges as a promising therapeutic agent due to its wide-ranging effects, including the modulation of microglia, reduction of neurodegeneration and vascular damage, and enhancement of visual function during the onset and early stages of diabetes (Fig. 6). This study fills a gap in the field by characterizing the effects of mFKN and sFKN. Additional studies suggest that the intracellular domain of FKN, found on the cytoplasmic tail (C-terminus; ctFKN) of the protein is also neuroprotective by inducing transcriptional regulation of genes essential for neuronal growth, differentiation, and maintenance [50]. Assessing the synergistic actions of sFKN and ctFKN in microglial and neuronal transcriptomic profile is open for further investigation. Our studies suggest that FKN isoforms can produce diverse outcomes in the context of neurodegenerative diseases that is likely linked to their ability to elicit different changes in microglial and neuronal phenotype [21, 34, 51]. This study furnishes evidence that mFKN and sFKN differentially regulate microglia-mediated inflammation and vascular injury, opening an avenue to test additional therapeutic modalities in humanized models. We demonstrate that increasing sFKN expression can help maintain neuronal densities, preserve optic nerve synaptic integrity, establish a homeostatic immune profile in the retina, and enhance visual acuity in DR models. These results expand our understanding of the processes initiating DR by suggesting that targeting inflammation can ameliorate visual function and that future research employing rAAV therapy is achievable.

### Supplementary Information

**Additional file 1: Fig. S1.** rAAV transduction occurs in neurons, without altering peripheral blood immune cell distributions. (**A**) Schematic presentation of vertical cross section of retinal layers with positions of vascular plexuses. (**B**) Gating strategy to identify CD45^Hi^CD11b^+^SSC^Hi^ neutrophils, CD45^Hi^CD11b^+^Ly6C^+^ inflammatory macrophages, CD45^Hi^CD11b^+^Ly6C^–^ tissue resident macrophages, CD45^Hi^CD11b^–^CD11c^+^ conventional dendritic cells, and CD45^Hi^CD11b^+^CD11c^+^ myeloid-derived dendritic cells in blood leukocytes. (**C–G**) Graphical representation of flow cytometric quantification of CD45^Hi^CD11b^+^SSC^Hi^ neutrophils (**C**), CD45^Hi^CD11b^+^Ly6C^–^ tissue-resident macrophages (**D**), CD45^Hi^CD11b^+^Ly6C^+^ inflammatory macrophages (**E**), CD45^Hi^CD11b^–^CD11c^+^ conventional dendritic cells (**F**), CD45^Hi^CD11b^+^CD11c^+^ myeloid-derived dendritic cells (**G**). Data are shown as mean ± SD, *n* = 4–6 mice per group, where each data point represents an individual mouse across five experiments. *p < 0.05, **p < 0.01, and ****p < 0.0001 using Student’s *t* test, Welch’s correction. **Fig. S2.** Intra-vitreal administration of rAAV does not further alter splenic immune cell distributions. (**A**) Gating strategy of splenocytes to identify CD45^Hi^CD11b^+^Ly6G^+^ granulocytes, CD45^Hi^CD11b^+^CD11c^–^Ly6C^+^ inflammatory macrophages with respective MHC-II antigen presentation, CD45^Hi^CD11b^+^CD11c^–^Ly6C^–^ tissue-resident macrophages, CD45^Hi^CD11b^–^CD11c^+^ conventional dendritic cells and CD45^Hi^CD11b^+^CD11c^+^ myeloid-derived dendritic cells (with respective MHC-II antigen presentation), CD45^Hi^CD11b^–^CD3^+^CD4^+^ T cells, CD45^Hi^CD11b^–^CD3^+^CD4^+^CD44^+^ activated T cells, CD45^Hi^CD11b^–^CD3^+^CD4^+^CD25^+^ T regulatory cells, CD45^Hi^CD11b^–^CD3^+^CD8^+^ T cells, CD45^Hi^CD11b^–^CD3^+^CD8^+^CD44^+^ activated T cells, and CD45^Hi^CD11b^–^CD3^+^CD8^+^CD25^+^ T regulatory cells. (**B–O**) Graphical representation of flow cytometric quantification of CD45^Hi^CD11b^+^Ly6G^+^ granulocytes (**B**), CD45^Hi^CD11b^+^CD11c^–^Ly6C^+^ inflammatory macrophages (**C**), CD45^Hi^CD11b^+^CD11c^–^Ly6C^+^MHCII^+^ inflammatory macrophages (**D**), CD45^Hi^CD11b^+^CD11c^–^Ly6C^–^ tissue-resident macrophages (**E**), CD45^Hi^CD11b^–^CD11c^+^ conventional dendritic cells (**F)**, CD45^Hi^CD11b^–^CD11c^+^MHCII^+^ activated conventional dendritic cells (**G**), CD45^Hi^CD11b^+^CD11c^+^ myeloid-derived dendritic cells (**H**), CD45^Hi^CD11b^+^CD11c^+^MHCII^+^ activated myeloid-derived dendritic cells (**I**), CD45^Hi^CD11b^–^CD3^+^CD4^+^ T cells (**J**), CD45^Hi^CD11b^–^CD3^+^CD4^+^CD44^+^ activated T cells (**K**), CD45^Hi^CD11b^–^CD3^+^CD4^+^CD25^+^ T regulatory cells (**L**), CD45^Hi^CD11b^–^CD3^+^CD8^+^ T cells (**M**), CD45^Hi^CD11b^–^CD3^+^CD8^+^CD44^+^ activated T cells (**N**), CD45^Hi^CD11b^–^CD3^+^CD8^+^CD25^+^ T regulatory cells (**O**). Data shown as mean ± SD, *n* = 5 mice per group, where each data point represents an individual mouse across two experimental replicates. *p < 0.05, **p < 0.01, and ****p < 0.0001 using Student’s *t* test, Welch’s correction. **Fig. S3.** Non-diabetic WT retinas display higher neuronal densities compared to FKN^KO^ tissues. (**A**) Confocal images of retinal tissues stained for NeuN (red) and Iba1 (green) (left panel), with representation of cellular tracing for transformation index analysis (*Inset*). Confocal images of retinal tissues stained for CD31 (red) and fibrinogen (white) (right panel) (Scale bar; 100 µm). (**B–E**) Quantification of retinal immunofluorescence analysis of NeuN^+^ cells/mm^3^ (**B**) and Iba1^+^ cells/mm^3^ (**C**), immunofluorescence analysis of CD31^+^ percent immunoreactive area (% IRA) (**D**) and fibrinogen^+^ percent immunoreactive area (% IRA) (**E**). Data shown as mean ± SD, *n* = 4–11 mice per group, where each dot represents an individual mouse across six experiments. *p < 0.05, **p < 0.01, ***p < 0.001 and ****p < 0.0001 using Student’s *t* test, Welch’s correction. **Fig. S4.** Intra-vitreal injection of rAAV–sFKN prevented axonal, SYP + loss, and astrogliosis in the diabetic optic nerve. (**A**) Confocal images of ND and diabetic optic nerves stained for visualization of axons (TUJ1, teal), synaptophysin (SYP, green), and astrocytes (GFAP, magenta) (Scale bar; 75 µm). (**B–D**) Quantification of the positive immunoreactive area (% IRA) for TUJ1^+^ (**B**), SYP^+^ (**C**), and GFAP^+^ (**D**). Data shown as mean ± SD, *n* = 4–6 mice per group, each data point represents an individual mouse across four experiments. *p < 0.05, **p < 0.01, and ****p < 0.0001 using Student’s *t* test, Welch’s correction. **Fig. S5.** Treatment with rAAV–sFKN shifts the microglial transcriptomic profile to a homeostatic state. (**A**) Gating strategy to identify CD11b^+^CD45^Lo^ microglia in brain and spinal cord tissues, CD11b^+^CD45^Hi^ infiltrating cells, CD11b^+^CD45^Lo^P2RY12^+^ homeostatic microglia, and CD11b^+^CD45^Lo^Ly6C^+^ inflammatory microglia. (**B–E**) Graphical representation of flow cytometric quantification of CD11b^+^CD45^Lo^ microglia in brain and spinal cord tissues (**B**), CD11b^+^CD45^Hi^ infiltrating cells (**C**), CD11b^+^CD45^Lo^P2RY12^+^ homeostatic microglia, (**D**), and CD11b^+^CD45^Lo^Ly6C^+^ inflammatory microglia (**E**). Data shown as mean ± SD, *n* = 4–6 mice per group, where each data point represents an individual mouse across four experiments. *p < 0.05 and **p < 0.01 using Student’s *t* test, Welch’s correction. **Fig. S6.** Transcriptome analyses of significantly expressed gene in response to rAAV–sFKN in the diabetic retina. Analysis of statistically significant DEGs associated with complement, cell death, and regulation of cell death (**A, E**), inflammation, (**B, F**), neuronal and vascular damage (**C, G**), and microglia activation and DR pathogenesis (**D, H**) of PBS-treated 4-wk D and 10-wk D mice compared their PBS-treated ND controls. Significant DEGs associated with complement, cell death, and regulation of cell death (**I, L**), inflammation (**J**), and neuronal and vascular damage (**K**) of 4-wk D and 10-wk D mice compared to diabetic rAAV–sFKN-treated groups. *n* = 4–5 mice per treatment group across two experiments. The level of significance in the DEGs associated with rAAV–sFKN treatment can be found in Table S4. **Fig. S7.** Bulk mRNA-seq transcriptome gene expression in rAAV–sFKN and rAAV–mFKN treated groups. Graphical representation and volcano plots showing all DEGs of each comparison group without set thresholds (i.e., log_2_ fold change of 1 and -1 with FDR *p* value of < 0.05), thresholds are distinguishably marked (dotted line panes and red colored points). Total DEGs of PBS-treated 4-wk D mice versus PBS-treated ND mice (**A, B**), PBS-treated 10-wk D mice versus PBS-treated ND mice (**C, D**), rAAV–mFKN-treated 4-wk D mice versus PBS-treated 4-wk D mice (**E, F**), rAAV–mFKN-treated 10-wk D mice versus PBS-treated 10-wk D mice (**G, H**), rAAV–sFKN-treated 4-wk D mice versus PBS-treated 4-wk D mice (**I, J**), and rAAV–sFKN-treated 10-wk D mice versus PBS-treated 10-wk D mice (**K, L**). *n* = 4–5 mice per treatment group across two experiments. The complete list of genes with set threshold can be found in Table S4. **Fig. S8.** Transcriptome gene expression in rAAV–mFKN treated groups. (**A–D**) Heat map gene expression of all up and down regulated DEGs associated with the complement pathway, cell death and cell death regulation (**A**), inflammatory cytokines and chemokines (**B**), vascular and neuronal damage (**C**) and microglia activation and DR pathogenesis (**D**). All heat maps compared PBS-treated 4-wk D and 10-wk D mice to PBS-treated ND controls, and PBS-treated 4-wk D and 10-wk D mice compared to rAAV–mFKN-treated mice. (**E–P**) Analysis of statistically significant DEGs associated with complement, cell death, and regulation of cell death (**E, I**), inflammation, (**F, J**), neuronal and vascular damage (**G, K**), and microglia activation and DR pathogenesis (**H, L**) of PBS-treated 4-wk D and 10-wk D mice compared their PBS-treated ND controls. Significant DEGs associated with complement, cell death, and regulation of cell death (**M**), inflammation (**N**), and neuronal and vascular damage (**O**), and microglia activation and DR pathogenesis (**P**) of 4-wk D mice compared to diabetic rAAV–mFKN-treated groups. *n* = 5 mice per treatment group across two experiments. The level of significance in the DEGs associated with rAAV–sFKN treatment can be found in Table S4. **Table S1.** Antibodies for immunohistochemistry analysis. **Table S2.** Combination of antibodies used for analysis.  **Table S3.** Antibodies for flow cytometry analysis.  **Table S4.** Level of significance in the differentially expression genes (DEGs) associated with rAAV–mFKN or rAAV–sFKN treatment.

## Data Availability

The data sets used and/or analyzed during the current study are available from the corresponding author on reasonable request.
