# Supplementary material for: Fractalkine isoforms differentially regulate microglia-mediated inflammation and enhance visual function in the diabetic retina
Source: J Neuroinflammation. 2024 Feb 4;21:42. doi: 10.1186/s12974-023-02983-8 (PMC10840196; doi:10.1186/s12974-023-02983-8)
Supplement: Supplementary file 1 — Additional file 1: Fig. S1. rAAV transduction occurs in neurons, without altering peripheral blood immune cell distributions. (A) Schematic presentation of vertical cross section of retinal layers with positions of vascular plexuses. (B) Gating strategy to identify CD45HiCD11b+SSCHi neutrophils, CD45HiCD11b+Ly6C+ inflammatory macrophages, CD45HiCD11b+Ly6C– tissue resident macrophages, CD45HiCD11b–CD11c+ conventional dendritic cells, and CD45HiCD11b+CD11c+ myeloid-derived dendritic cells in blood leukocytes. (C–G) Graphical representation of flow cytometric quantification of CD45HiCD11b+SSCHi neutrophils (C), CD45HiCD11b+Ly6C– tissue-resident macrophages (D), CD45HiCD11b+Ly6C+ inflammatory macrophages (E), CD45HiCD11b–CD11c+ conventional dendritic cells (F), CD45HiCD11b+CD11c+ myeloid-derived dendritic cells (G). Data are shown as mean ± SD, n = 4–6 mice per group, where each data point represents an individual mouse across five experiments. *p < 0.05, **p < 0.01, and ****p < 0.0001 using Student’s t test, Welch’s correction. Fig. S2. Intra-vitreal administration of rAAV does not further alter splenic immune cell distributions. (A) Gating strategy of splenocytes to identify CD45HiCD11b+Ly6G+ granulocytes, CD45HiCD11b+CD11c–Ly6C+ inflammatory macrophages with respective MHC-II antigen presentation, CD45HiCD11b+CD11c–Ly6C– tissue-resident macrophages, CD45HiCD11b–CD11c+ conventional dendritic cells and CD45HiCD11b+CD11c+ myeloid-derived dendritic cells (with respective MHC-II antigen presentation), CD45HiCD11b–CD3+CD4+ T cells, CD45HiCD11b–CD3+CD4+CD44+ activated T cells, CD45HiCD11b–CD3+CD4+CD25+ T regulatory cells, CD45HiCD11b–CD3+CD8+ T cells, CD45HiCD11b–CD3+CD8+CD44+ activated T cells, and CD45HiCD11b–CD3+CD8+CD25+ T regulatory cells. (B–O) Graphical representation of flow cytometric quantification of CD45HiCD11b+Ly6G+ granulocytes (B), CD45HiCD11b+CD11c–Ly6C+ inflammatory macrophages (C), CD45HiCD11b+CD11c–Ly6C+MHCII+ inflammatory macrophages (D), CD45HiCD11b+CD1 [file 12974_2023_2983_MOESM1_ESM.docx]

**Supplementary Figures**

**Fig. S1. rAAV transduction occurs in neurons, without altering peripheral blood immune cell distributions.** (**A**) Schematic presentation of vertical cross-section of retinal layers with positions of vascular plexuses. (**B**) Gating strategy to identify CD45^Hi^CD11b^+^SSC^Hi^ neutrophils, CD45^Hi^CD11b^+^Ly6C^+^ inflammatory macrophages, CD45^Hi^CD11b^+^Ly6C^–^ tissue resident macrophages, CD45^Hi^CD11b^–^CD11c^+^ conventional dendritic cells, and CD45^Hi^CD11b^+^CD11c^+^ myeloid-derived dendritic cells in blood leukocytes. (**C-G**) Graphical representation of flow cytometric quantification of CD45^Hi^CD11b^+^SSC^Hi^ neutrophils (**C**), CD45^Hi^CD11b^+^Ly6C^–^ tissue-resident macrophages (**D**), CD45^Hi^CD11b^+^Ly6C^+^ inflammatory macrophages (**E**), CD45^Hi^CD11b^–^CD11c^+^ conventional dendritic cells (**F**), CD45^Hi^CD11b^+^CD11c^+^ myeloid-derived dendritic cells (**G**). Data is shown as mean ± SD, *n* = 4-6 mice per group where each data point represents an individual mouse across five experiments. *p < 0.05, **p < 0.01, and ****p < 0.0001 using Student’s *t* test, Welch’s correction.


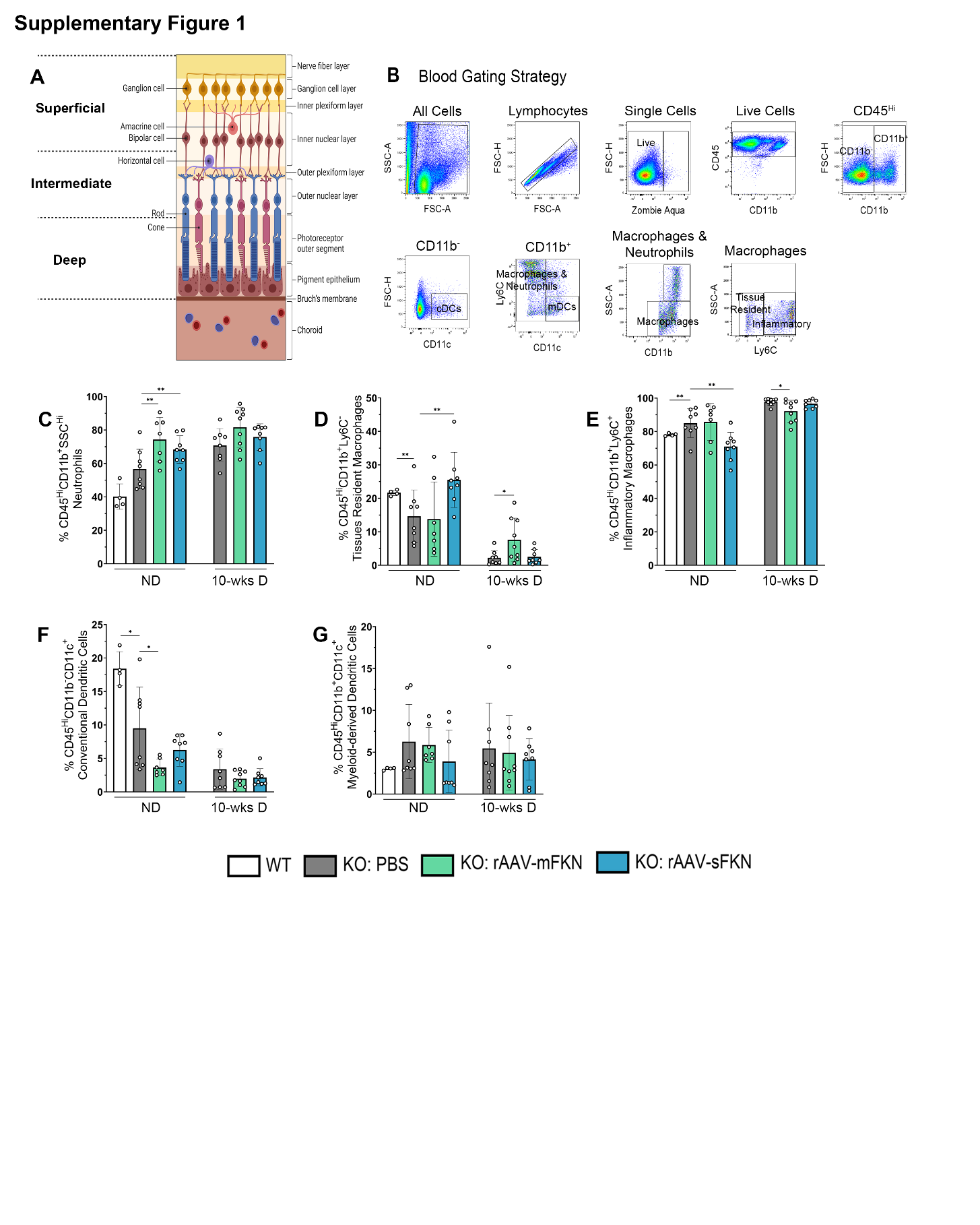


**Fig. S2. Intra-vitreal administration of rAAV does not further alter splenic immune cell distributions.** Figure legend on next page.


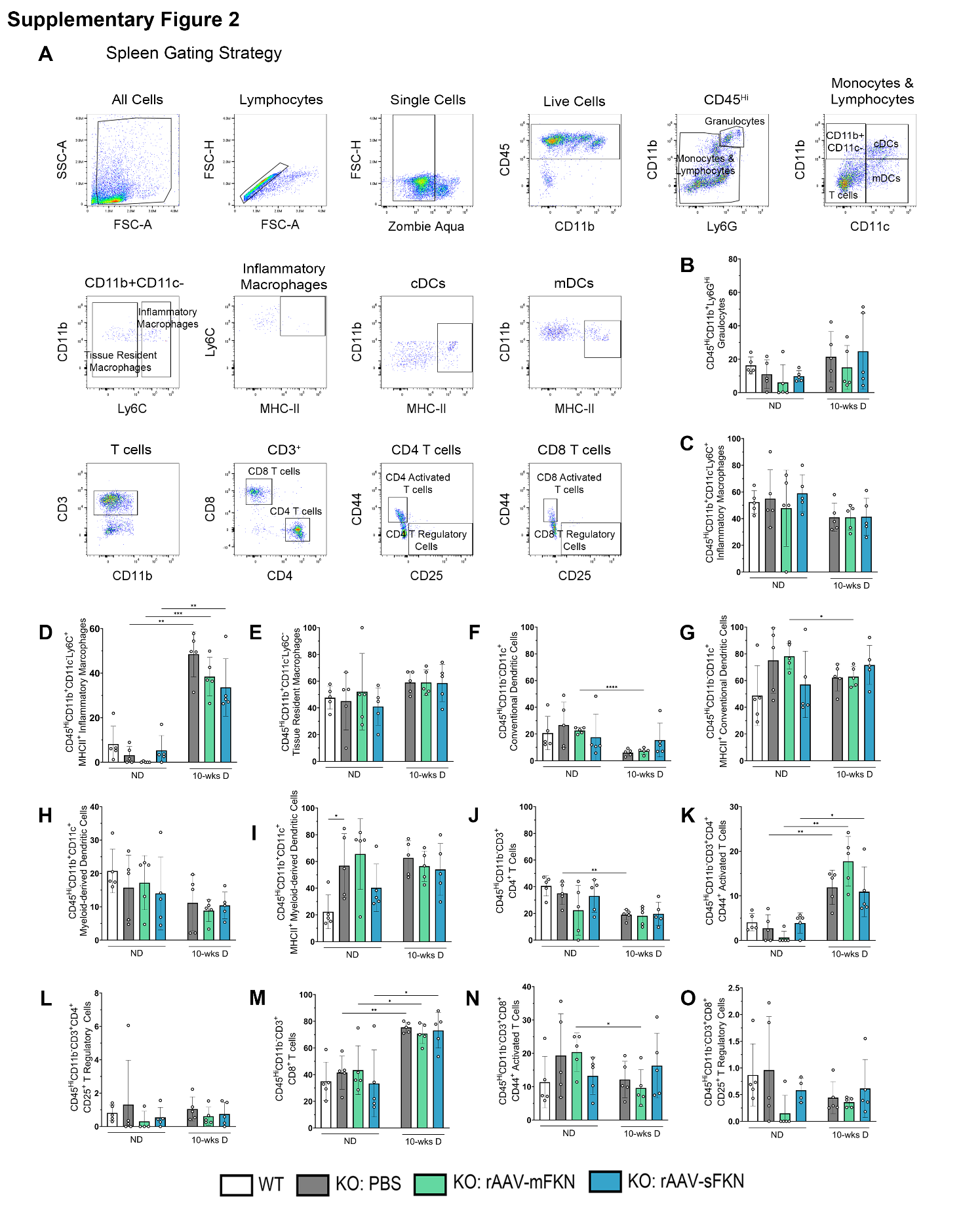


**Fig. S2. Intra-vitreal administration of rAAV does not further alter splenic immune cell distributions.** (**A**) Gating strategy of splenocytes to identify CD45^Hi^CD11b^+^Ly6G^+^ granulocytes, CD45^Hi^CD11b^+^CD11c^–^Ly6C^+^ inflammatory macrophages with respective MHC-II antigen presentation, CD45^Hi^CD11b^+^CD11c^–^Ly6C^–^ tissue-resident macrophages, CD45^Hi^CD11b^–^CD11c^+^ conventional dendritic cells and CD45^Hi^CD11b^+^CD11c^+^ myeloid-derived dendritic cells (with respective MHC-II antigen presentation), CD45^Hi^CD11b^–^CD3^+^CD4^+^ T cells, CD45^Hi^CD11b^–^CD3^+^CD4^+^CD44^+^ activated T cells, CD45^Hi^CD11b^–^CD3^+^CD4^+^CD25^+^ T regulatory cells, CD45^Hi^CD11b^–^CD3^+^CD8^+^ T cells, CD45^Hi^CD11b^–^CD3^+^CD8^+^CD44^+^ activated T cells, and CD45^Hi^CD11b^–^CD3^+^CD8^+^CD25^+^ T regulatory cells. (**B-O**) Graphical representation of flow cytometric quantification of CD45^Hi^CD11b^+^Ly6G^+^ granulocytes (**B**), CD45^Hi^CD11b^+^CD11c^–^Ly6C^+^ inflammatory macrophages (**C**), CD45^Hi^CD11b^+^CD11c^–^Ly6C^+^MHCII^+^ inflammatory macrophages (**D**), CD45^Hi^CD11b^+^CD11c^–^Ly6C^–^ tissue-resident macrophages (**E**), CD45^Hi^CD11b^–^CD11c^+^ conventional dendritic cells (**F)**, CD45^Hi^CD11b^–^CD11c^+^MHCII^+^ activated conventional dendritic cells (**G**), CD45^Hi^CD11b^+^CD11c^+^ myeloid-derived dendritic cells (**H**), CD45^Hi^CD11b^+^CD11c^+^MHCII^+^ activated myeloid-derived dendritic cells (**I**), CD45^Hi^CD11b^–^CD3^+^CD4^+^ T cells (**J**), CD45^Hi^CD11b^–^CD3^+^CD4^+^CD44^+^ activated T cells (**K**), CD45^Hi^CD11b^–^CD3^+^CD4^+^CD25^+^ T regulatory cells (**L**), CD45^Hi^CD11b^–^CD3^+^CD8^+^ T cells (**M**), CD45^Hi^CD11b^–^CD3^+^CD8^+^CD44^+^ activated T cells (**N**), CD45^Hi^CD11b^–^CD3^+^CD8^+^CD25^+^ T regulatory cells (**O**). Data shown as mean ± SD, *n* = 5 mice per group where each data point represents an individual mouse across two experimental replicates. *p < 0.05, **p < 0.01, and ****p < 0.0001 using Student’s *t* test, Welch’s correction.

**Fig. S3. Non-diabetic WT retinas display higher neuronal densities compared to FKN^KO^ tissues.** (**A**) Confocal images of retinal tissues stained for NeuN (red) and Iba1 (green) (left panel), with representation of cellular tracing for transformation index analysis (Inset). Confocal images of retinal tissues stained for CD31 (red) and fibrinogen (white) (right panel) (Scale bar; 100 µm). (**B-E**) Quantification of retinal immunofluorescence analysis of NeuN^+^ cells/mm^3^ (**B**) and Iba1^+^ cells/mm^3^ (**C**), immunofluorescence analysis of CD31^+^ percent immunoreactive area (% IRA) (**D**) and fibrinogen^+^ percent immunoreactive area (% IRA) (**E**). Data shown as mean ± SD, *n* = 4-11 mice per group where each dot represents an individual mouse across six experiments. *p < 0.05, **p < 0.01, ***p < 0.001 and ****p < 0.0001 using Student’s *t* test, Welch’s correction.


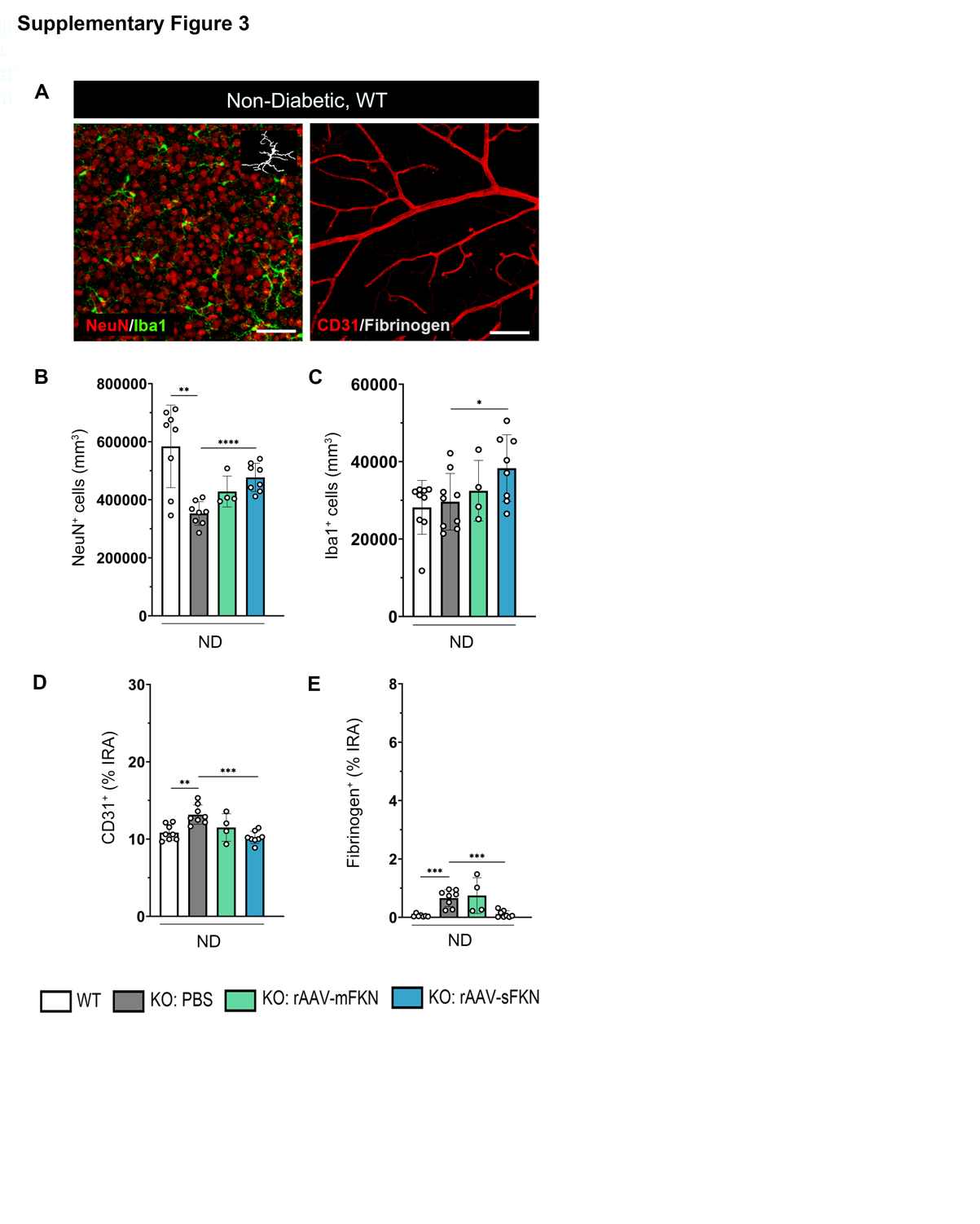


**SUPPLEMENTARY TABLES**

**Fig. S4. Intra-vitreal injection of rAAV-sFKN prevented axonal, SYP^+^ loss, and astrogliosis in the diabetic optic nerve.** (**A**) Confocal images of ND and diabetic optic nerves stained for visualization of axons (TUJ1, teal), synaptophysin (SYP, green), and astrocytes (GFAP, magenta) (Scale bar; 75 µm). (**B-D**) Quantification of the positive immunoreactive area (% IRA) for TUJ1^+^ (**B**), SYP^+^ (**C**), and GFAP^+^ (**D**). Data shown as mean ± SD, *n* = 4-6 mice per group, each data point represents an individual mouse across four experiments. *p < 0.05, **p < 0.01, and ****p < 0.0001 using Student’s *t* test, Welch’s correction.


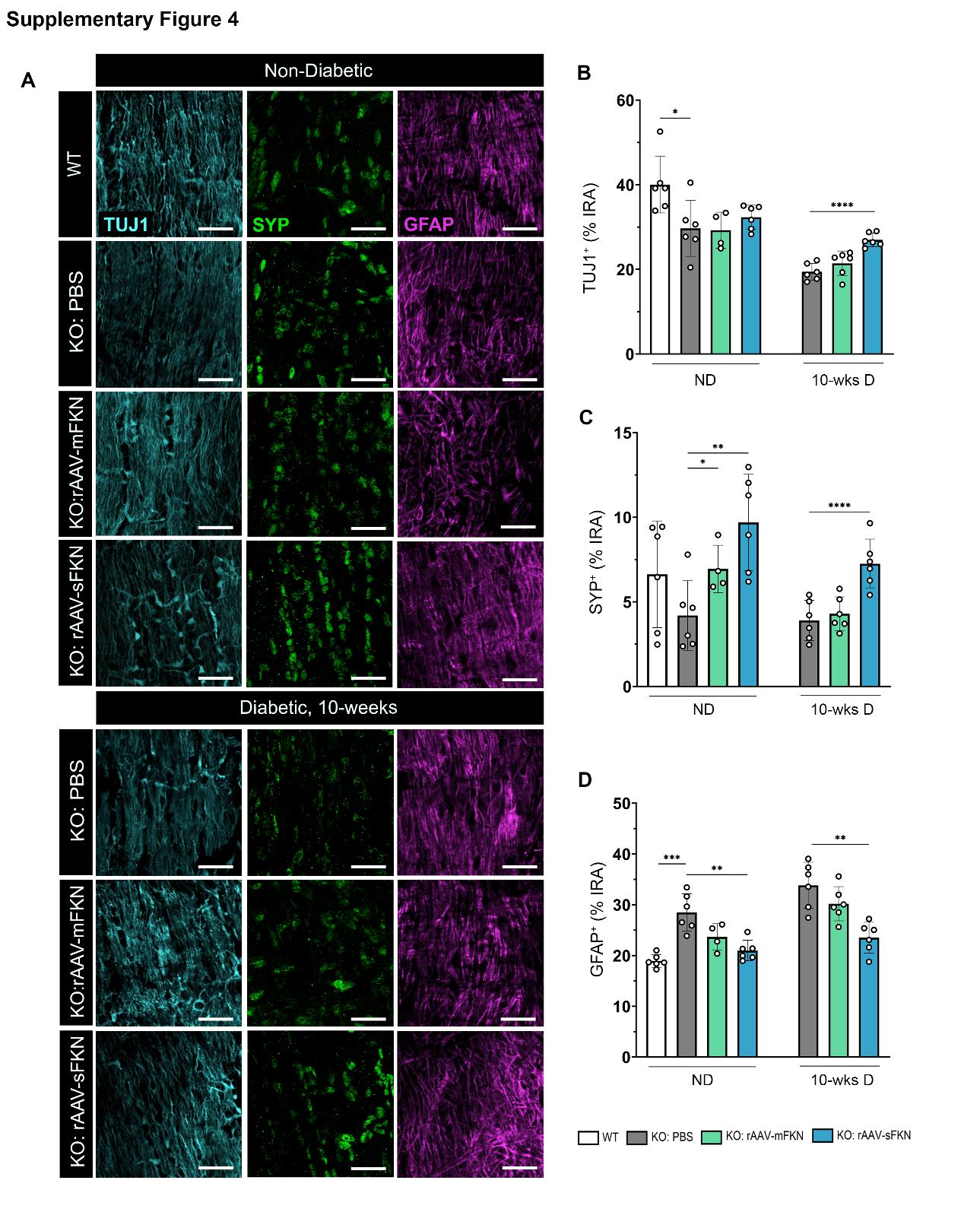


**Fig. S5. Treatment with rAAV-sFKN shifts the microglial transcriptomic profile to a homeostatic state.** (**A**) Gating strategy to identify CD11b^+^CD45^Lo^ microglia in brain and spinal cord tissues, CD11b^+^CD45^Hi^ infiltrating cells, CD11b^+^CD45^Lo^P2RY12^+^ homeostatic microglia, and CD11b^+^CD45^Lo^Ly6C^+^ inflammatory microglia. (**B-E**) Graphical representation of flow cytometric quantification of CD11b^+^CD45^Lo^ microglia in brain and spinal cord tissues (**B**), CD11b^+^CD45^Hi^ infiltrating cells (**C**), CD11b^+^CD45^Lo^P2RY12^+^ homeostatic microglia, (**D**), and CD11b^+^CD45^Lo^Ly6C^+^ inflammatory microglia (**E**). Data shown as mean ± SD, *n* = 4-6 mice per group where each data point represents an individual mouse across four experiments. *p < 0.05 and **p < 0.01 using Student’s *t* test, Welch’s correction.


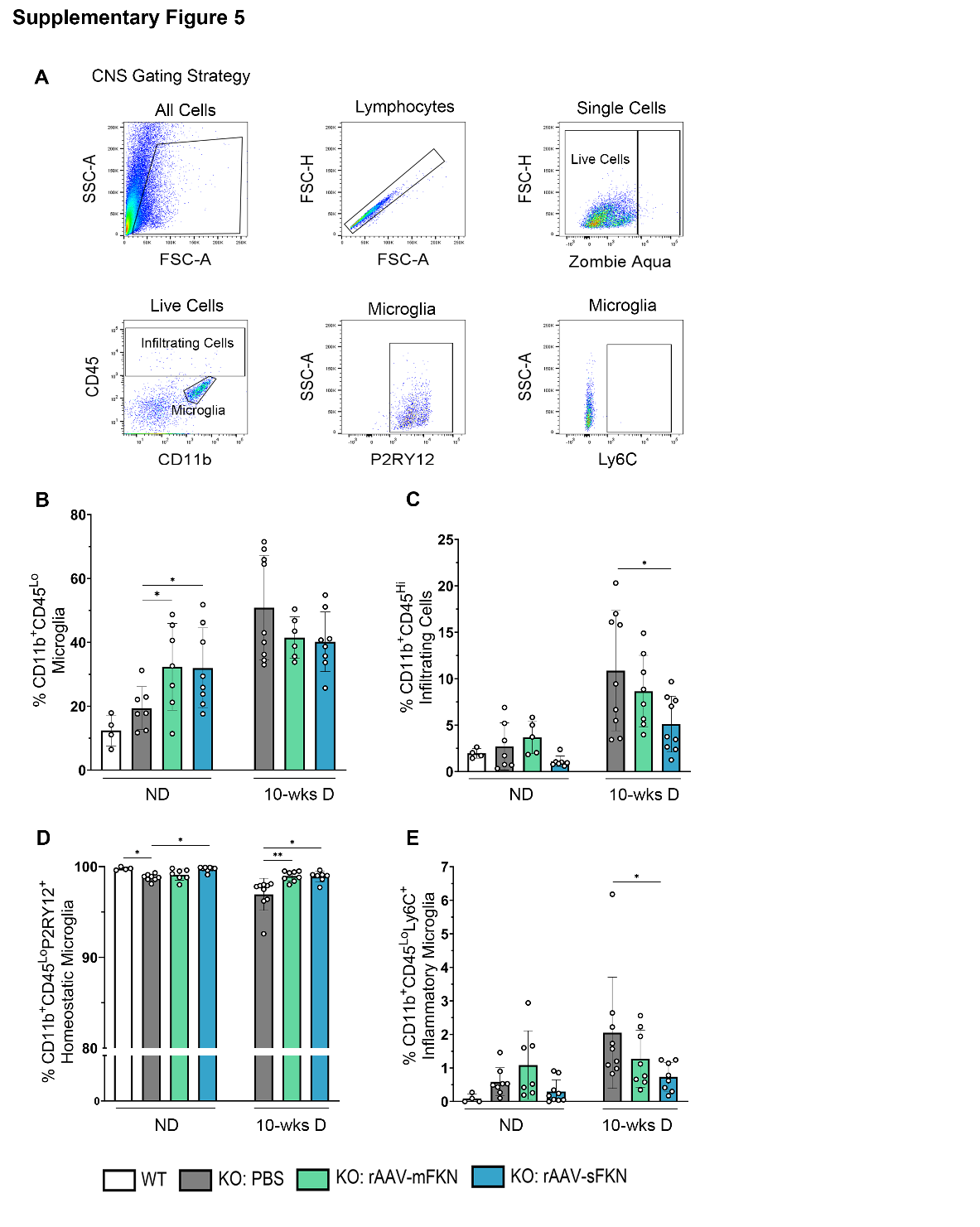


**Fig. S6. Transcriptome analyses of significantly expressed gene in response to rAAV-sFKN in the diabetic retina.** Analysis of statistically significant DEGs associated with complement, cell death, and regulation of cell death (**A, E**), inflammation, (**B, F**), neuronal and vascular damage (**C, G**), and microglia activation and DR pathogenesis (**D, H**) of PBS-treated 4-wks D and 10-wks D mice compared their PBS-treated ND controls. Significant DEGs associated with complement, cell death, and regulation of cell death (**I, L**), inflammation (**J**), and neuronal and vascular damage (**K**) of 4-wks D and 10-wks D mice compared to diabetic rAAV-sFKN-treated groups. *n* = 4-5 mice per treatment group across two experiments. The level of significance in the DEGs associated with rAAV-sFKN treatment can be found in Supplementary Table 4.


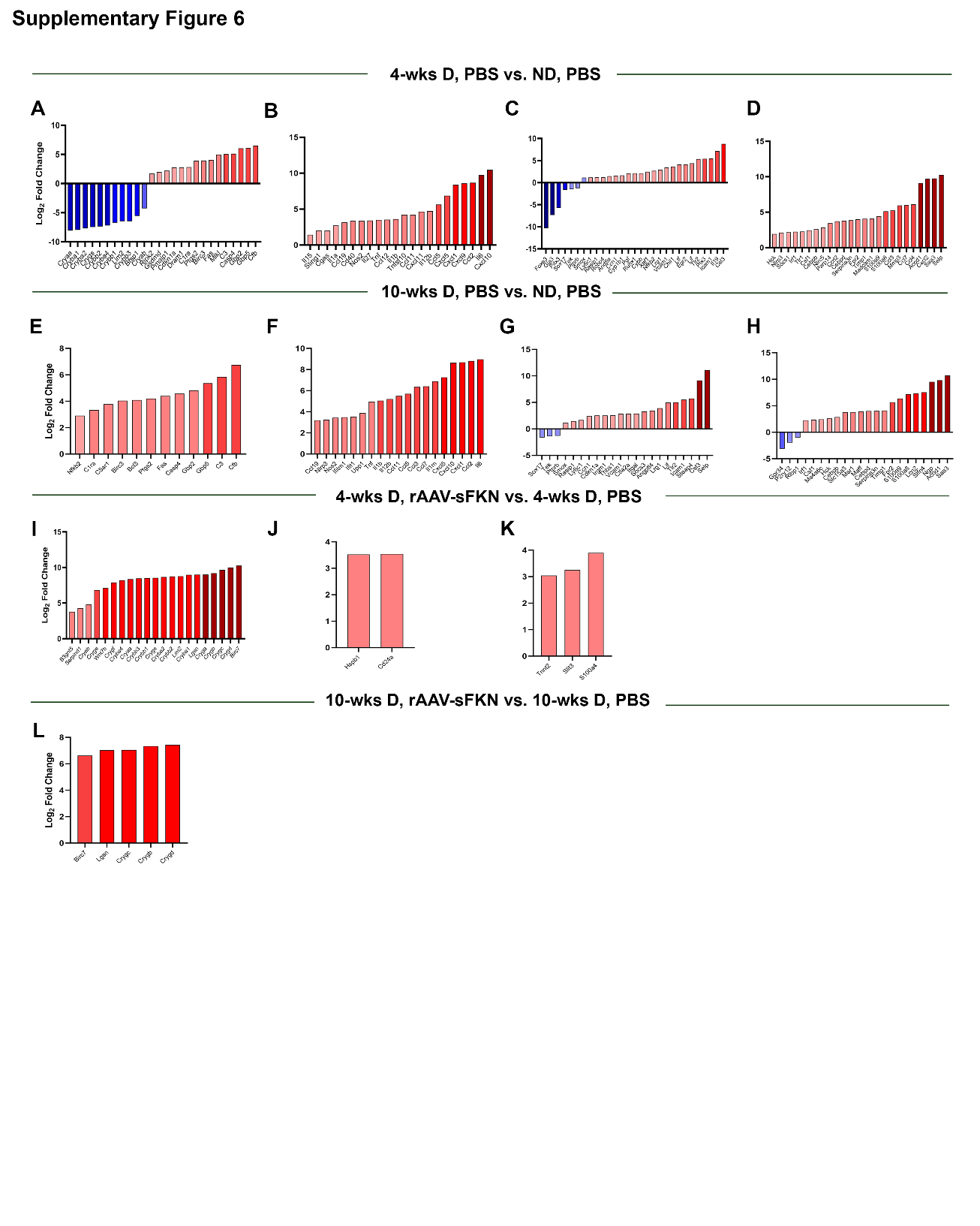


**Fig. S7. Bulk mRNAseq transcriptome gene expression in rAAV-sFKN and rAAV-mFKN treated groups.** Graphical representation and volcano plots showing all DEGs of each comparison group without set thresholds (i.e. log_2_ fold change of 1 and -1 with FDR P value of < 0.05), thresholds are distinguishably marked (dotted line panes and red colored points). Total DEGs of PBS-treated 4-wks D mice versus PBS-treated ND mice (**A-B**), PBS-treated 10-wks D mice versus PBS-treated ND mice (**C-D**), rAAV-mFKN-treated 4-wks D mice versus PBS-treated 4-wks D mice (**E-F**), rAAV-mFKN-treated 10-wks D mice versus PBS-treated 10-wks D mice (**G-H**), rAAV-sFKN-treated 4-wks D mice versus PBS-treated 4-wks D mice (**I-J**), and rAAV-sFKN-treated 10-wks D mice versus PBS-treated 10-wks D mice (**K-L**). *n* = 4-5 mice per treatment group across two experiments. The complete list of genes with set threshold can be found in Supplementary Table 4.


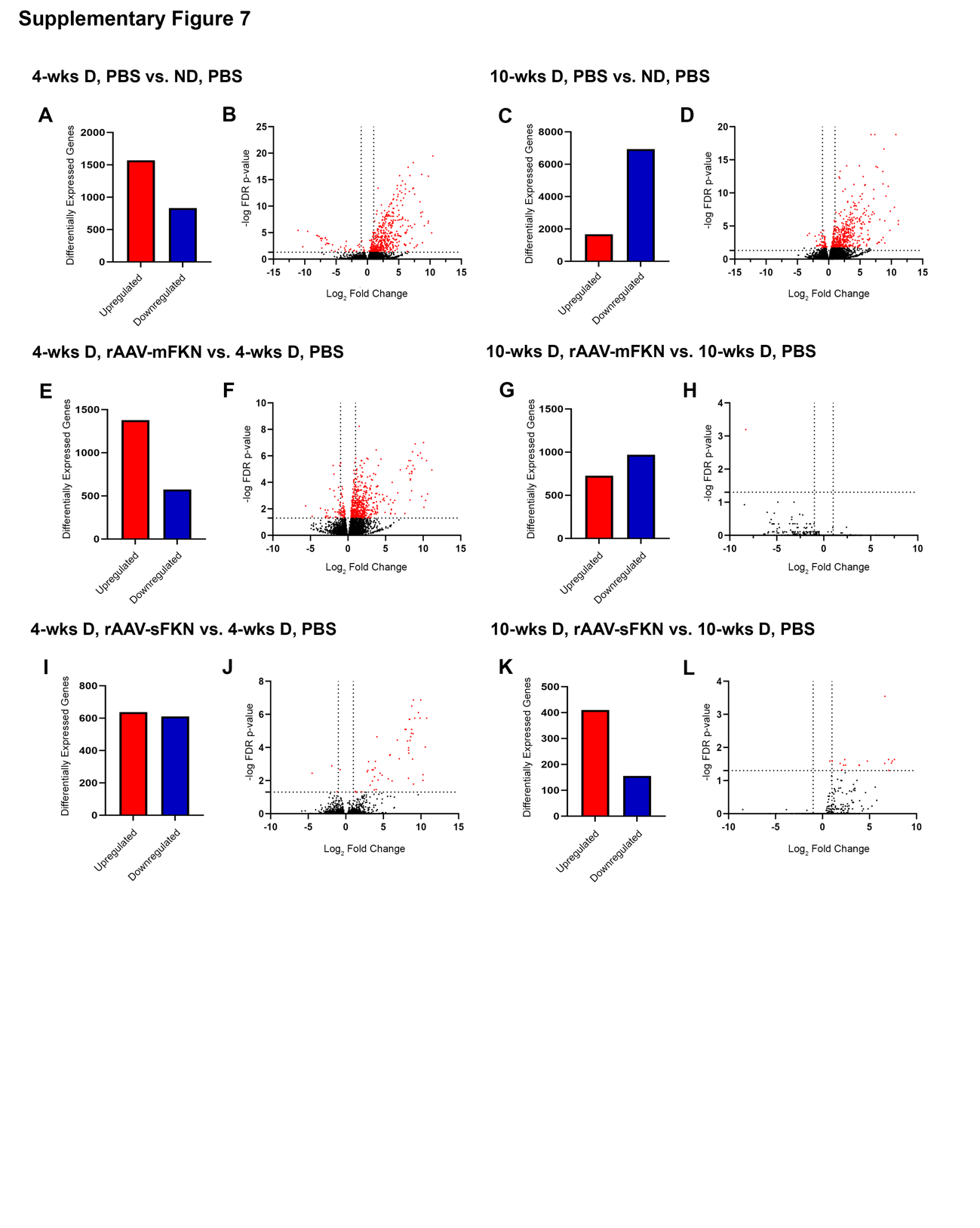


**Fig. S8. Transcriptome gene expression in rAAV-mFKN treated groups.** (**A-D**) Heat map gene expression of all up and down regulated DEGs associated with the complement pathway, cell death and cell death regulation (**A**), inflammatory cytokines and chemokines (**B**), vascular and neuronal damage (**C**) and microglia activation and DR pathogenesis (**D**). All heat maps compared PBS-treated 4-wks D and 10-wks D mice to PBS-treated ND controls, and PBS-treated 4-wks D and 10-wks D mice compared to rAAV-mFKN-treated mice. (**E-P**) Analysis of statistically significant DEGs associated with complement, cell death, and regulation of cell death (**E, I**), inflammation, (**F, J**), neuronal and vascular damage (**G, K**), and microglia activation and DR pathogenesis (**H, L**) of PBS-treated 4-wks D and 10-wks D mice compared their PBS-treated ND controls. Significant DEGs associated with complement, cell death, and regulation of cell death (**M**), inflammation (**N**), and neuronal and vascular damage (**O**), and microglia activation and DR pathogenesis (**P**) of 4-wks D mice compared to diabetic rAAV-mFKN-treated groups. *n* = 5 mice per treatment group across two experiments. The level of significance in the DEGs associated with rAAV-sFKN treatment can be found in Supplementary Table 4.


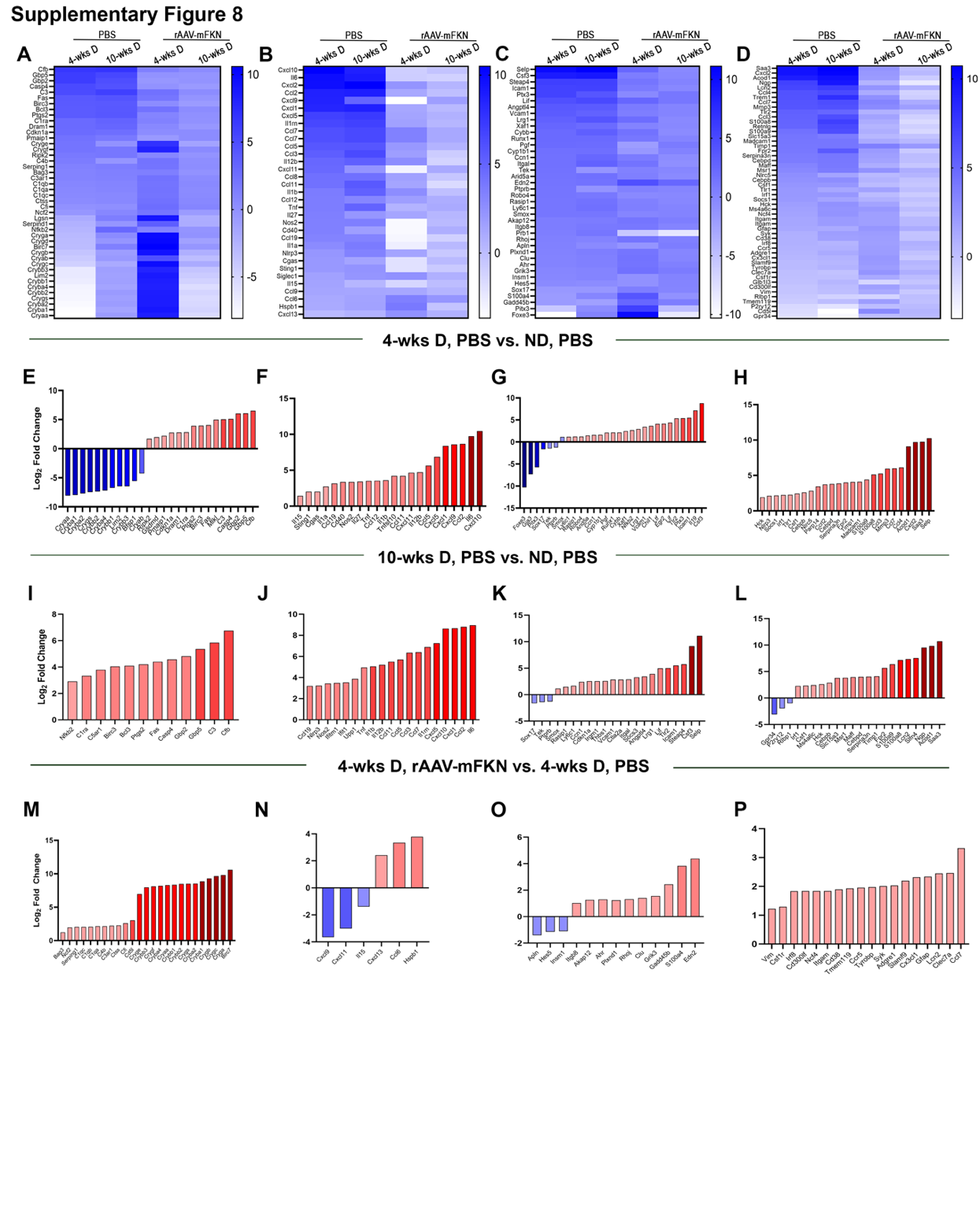


**Supplementary Table 1. Antibodies for immunohistochemistry analysis**

| **Target antigen (Clone)** | **Company** | **RRID** | **Dilution** |
| --- | --- | --- | --- |
| **Primary antibodies** | | | |
| Rabbit anti-ionized calcium binding adaptor molecule-1 (Iba1) | FUJIFILM-Wako | AB_839504 | 1:3000 |
| Mouse anti-neuronal nuclei (NeuN) | Millipore | AB_2298772 | 1:4000 |
| Rabbit anti-synaptophysin (SYP) | Millipore | AB_570874 | 1:500 |
| Mouse anti-tubulin beta 3 (TUJ1) | Biolegend | AB_10063408 | 1:1000 |
| Rat anti-glial fibrillary acidic protein (GFAP) | Invitrogen | AB_2532994 | 1:4000 |
| Rat anti-platelet endothelial cell adhesion molecule (PECAM-1/CD31) | BD Biosciences | AB_393571 | 1:500 |
| Rabbit anti-fibrinogen | Agilent | AB_578481 | 1:2000 |
| Rat anti-fractalkine | R&D Systems | AB_2087139 | 1:200 |
| **Secondary antibodies** | | | |
| Goat anti-mouse Cy3 | Jackson ImmunoResearch Laboratories, Inc | AB_2338709 | 1:1000 |
| Goat anti-rabbit 488 |  | AB_2338058 | 1:1000 |
| Goat anti-rat AlexaFluor 647 |  | AB_2338394 | 1:1000 |
| Goat anti-rabbit Cy3 |  | AB_2338006 | 1:1000 |
| Donkey anti-rat Alexa Fluor 488 |  | AB_2340684 | 1:1000 |
| Streptavidin Cy3 |  | AB_2337244 | 1:1000 |
| Streptavidin Cy2 |  | AB_2337246 | 1:1000 |
| Biotin goat anti-rabbit | Vector Laboratories | AB_2313606 | 1:1000 |
| Biotin goat anti-mouse |  | AB_2336171 | 1:1000 |

**Supplementary Table 2. Combination of antibodies used for analysis**

| **Target antigen** | **Primary antibody** | **Secondary antibody** |
| --- | --- | --- |
| Iba1 | Rabbit anti-Iba1 | Goat anti-rabbit 488 |
| NeuN | Mouse anti- NeuN | Biotin goat anti-mouse/Streptavidin Cy3 |
| SYP | Rabbit anti- SYP | Biotin goat anti-rabbit/Streptavidin Cy2 |
| TUJ1 | Mouse anti-TUJ1 | Goat anti-mouse Cy3 |
| GFAP | Rat anti-GFAP | Goat anti-rat AlexaFluor 647 |
| PECAM-1/CD31 | Rat anti-CD31 | Goat anti-rat AlexaFluor 647 |
| Fibrinogen | Rabbit anti-fibrinogen | Goat anti-rabbit Cy3 |
| Fractalkine | Rat anti-fractalkine | Donkey anti-rat 488 |

**Supplementary Table 3. Antibodies for flow cytometry analysis**

| **Target antigen (Clone)** | **Company** | **RRID** | **Dilution** |
| --- | --- | --- | --- |
| CD11b PE-CF594 (M1/70) | BD Bioscience | AB_11154422 | 1:100 |
| CD45 Pacific Blue (30-F11) | Invitrogen | AB_1518806 | 1:100 |
| CD11c PE-Cy7 (N418) | Invitrogen | AB_465552 | 1:50 |
| P2RY12 APC (S16007D) | Biolegend | AB_2721469 | 1:100 |
| Ly6C PE (HK1.4) | Biolegend | AB_1186132 | 1:30 |
| CD3 FITC (145-2C11) | BD Bioscience | AB_394595 | 1:200 |
| CD4 BV785 (GK1.5) | Biolegend | AB_2565843 | 1:200 |
| Ly6G PerCP Cy5.5 (1A8) | Biolegend | AB_1877272 | 1:200 |
| CD8 APC (53-6.7) | Biolegend | AB_312751 | 1:200 |
| CD25 APC-Cy7 (3C7) | Biolegend | AB_2650982 | 1:100 |
| CD44 PerCP (IM7) | Leinco Technologies | AB_2828971 | 1:400 |
| MHC-II BV421 M5/114.15.2 | BD Bioscience | AB_2716857 | 1:400 |

**Supplementary Table 4. Level of significance in the differentially expression genes (DEGs) associated with rAAV-mFKN or rAAV-sFKN treatment**

| **Gene** | **Name** | **Fold Change (Log_2_)** | **FDR p-value (p < 0.05)** |
| --- | --- | --- | --- |
| 4-wks D, PBS vs. ND, PBS | | | |
| Genes involved in complement and cell death | | | |
| Cryaa | Crystallin alpha A | -8.078678437 | 3.41E-05 |
| Cryba1 | Crystallin beta A1 | -7.951066309 | 4.01E-05 |
| Cryba2 | Crystallin beta A2 | -7.706805988 | 5.24E-05 |
| Crygs | Crystallin gamma S | -7.469950941 | 1.27E-04 |
| Crybb2 | Crystallin beta B2 | -7.369332871 | 1.14E-04 |
| Cryba4 | Crystallin beta A4 | -7.202859462 | 1.31E-04 |
| Crybb1 | Crystallin beta B1 | -6.729579401 | 1.09E-04 |
| Lim2 | Lens intrinsic membrane protein 2 | -6.477192682 | 9.68E-04 |
| Crybb3 | Crystallin beta B3 | -6.476686713 | 2.24E-04 |
| Bfsp1 | Beaded filament structural protein 1 | -5.551566742 | 1.96E-03 |
| Cryab | Crystallin alpha B | -4.257292591 | 5.16E-03 |
| Ripk2 | Receptor interacting serine/threonine kinase 2 | 1.7297 | 5.27E-03 |
| Gsdmd | Gasdermin D | 1.9673 | 3.01E-04 |
| Pmaip1 | Phorbol-12-myristate-13-acetate-induced protein 1 | 2.2225 | 3.14E-04 |
| Cdkn1a | Cyclin dependent kinase inhibitor 1A | 2.7485 | 7.03E-10 |
| Dram1 | DNA damage regulated autophagy modulator 1 | 2.7967 | 2.12E-04 |
| C1ra | Complement C1ra | 2.814791644 | 7.42E-05 |
| Ptgs2 | Prostaglandin-endoperoxide synthase 2 | 3.9219 | 2.18E-07 |
| Birc3 | Baculoviral IAP repeat containing 3 | 3.9651 | 5.41E-07 |
| Fas | Fas cell surface death receptor | 4.063679758 | 1.23E-07 |
| Mlkl | Mixed lineage kinase domain like pseudokinase | 4.986 | 5.93E-10 |
| C3 | Complement C3 | 5.054798676 | 4.25E-09 |
| Casp4 | Caspase 4 | 5.1286 | 3.58E-08 |
| Gbp2 | Guanylate binding protein 2 | 6.0585 | 6.93E-15 |
| Gbp5 | Guanylate binding protein 5 | 6.0993 | 5.90E-14 |
| Cfb | Complement factor B | 6.502550547 | 4.53E-18 |
| Genes involved in inflammation | | | |
| Il15 | Interleukin 15 | 1.44 | 0.04195736 |
| Sting1 | Stimulator of interferon response cGAMP interactor 1 | 2.00 | 5.49E-03 |
| Cgas | Cyclic GMP-AMP synthase | 2.01 | 5.40E-03 |
| Il1a | Interleukin 1 alpha | 2.73 | 0.028304245 |
| Ccl19 | C-C motif chemokine ligand 19 | 3.16 | 1.39E-04 |
| Cd40 | CD40 molecule | 3.36 | 3.95E-05 |
| Nos2 | Nitric oxide synthase 2 | 3.36 | 2.57E-04 |
| Il27 | Interleukin 27 | 3.41 | 0.040316507 |
| Tnf | Tumor necrosis factor | 3.52 | 1.52E-03 |
| Ccl12 | C-C motif chemokine ligand 12 | 3.52 | 2.13E-04 |
| Il1b | Interleukin 1 beta | 3.60 | 7.72E-03 |
| Tnfsf10 | TNF superfamily member 10 | 4.21 | 2.73E-10 |
| Ccl11 | C-C motif chemokine ligand 11 | 4.21 | 1.58E-04 |
| Cxcl11 | C-X-C motif chemokine ligand 11 | 4.65 | 1.88E-04 |
| Il12b | Interleukin 12 beta | 4.73 | 7.22E-04 |
| Ccl5 | C-C motif chemokine ligand 5 | 5.64 | 2.39E-08 |
| Cxcl5 | C-X-C motif chemokine ligand 5 | 6.86 | 5.35E-07 |
| Cxcl1 | C-X-C motif chemokine ligand 1 | 8.38 | 1.51E-11 |
| Cxcl9 | C-X-C motif chemokine ligand 9 | 8.58 | 9.27E-09 |
| Ccl2 | C-C motif chemokine ligand 2 | 8.65 | 1.05E-16 |
| Il6 | Interleukin 6 | 9.73 | 7.04E-08 |
| Cxcl10 | C-X-C motif chemokine ligand 10 | 10.45 | 3.37E-20 |
| Genes involved in vascular and neuronal damage | | | |
| Foxe3 | Forkhead Box E3 | -10.3182 | 4.52E-03 |
| Gja3 | Gap Junction Protein Alpha 3 | -7.34898 | 3.15E-04 |
| Pitx3 | Paired Like Homeodomain 3 | -5.7318 | 0.022723983 |
| Sox17 | SRY-Box Transcription Factor 17 | -1.68691 | 0.03207622 |
| Tek | TEK receptor tyrosine kinase | -1.5027 | 2.54E-03 |
| Ptprb | Protein Tyrosine Phosphatase Receptor Type B | -1.26004 | 1.56E-03 |
| Smox | Spermine Oxidase | 1.1352 | 6.78E-08 |
| Ly6c1 | Lymphocyte antigen 6 family member C1 | 1.1478 | 1.15E-03 |
| Rasip1 | Ras Interacting Protein 1 | 1.1896 | 1.24E-03 |
| Robo4 | Roundabout guidance receptor 4 | 1.2101 | 3.46E-03 |
| Arid5a | AT-rich interaction domain 5A | 1.4484 | 2.19E-03 |
| Ccn1 | Cellular communication network factor 1 | 1.5798 | 0.030122526 |
| Cyp1b1 | Cytochrome P450 family 1 subfamily B member 1 | 1.6237 | 5.49E-03 |
| Pgf | Placental growth factor | 2.1159 | 0.016898246 |
| Runx1 | RUNX family transcription factor 1 | 2.1516 | 0.010869664 |
| Cybb | Cytochrome b-245 beta chain | 2.1661 | 6.20E-03 |
| Xaf1 | XIAP associated factor 1 | 2.4766 | 9.69E-09 |
| Nfkb2 | Nuclear factor kappa B subunit 2 | 2.6776 | 1.64E-05 |
| Lrg1 | Leucine rich alpha-2-glycoprotein 1 | 2.9419 | 7.20E-03 |
| Vcam1 | Vascular cell adhesion molecule 1 | 3.4267 | 4.80E-09 |
| Chil1 | Chitinase-3-like protein 1 | 3.6459 | 3.35E-07 |
| Ltf | Lactotransferrin | 4.0859 | 7.03E-04 |
| Egr2 | Early growth response 2 | 4.136 | 3.39E-03 |
| Lif | LIF interleukin 6 family cytokine | 4.3798 | 3.11E-05 |
| Tlr2 | Toll like receptor 2 | 5.3339 | 8.90E-12 |
| Ptx3 | Pentraxin 3 | 5.4137 | 3.31E-08 |
| Icam1 | Intercellular adhesion molecule 1 | 5.4804 | 2.83E-13 |
| Il19 | Interleukin 19 | 7.1449 | 1.29E-04 |
| Csf3 | Colony stimulating factor 3 | 8.7795 | 1.28E-09 |
| Genes involved in microglia activation and DR pathogenesis | | | |
| Hck | HCK proto-oncogene, Src family tyrosine kinase | 1.9087 | 0.01878196 |
| Nlrp3 | NLR family pyrin domain containing 3 | 2.1141 | 0.030183488 |
| Socs1 | Suppressor of cytokine signaling 1 | 2.1547 | 1.04E-03 |
| Irf1 | Interferon regulatory factor 1 | 2.2358 | 1.09E-05 |
| Tlr1 | Toll like receptor 1 | 2.2546 | 0.021823376 |
| Csf1 | Colony stimulating factor 1 | 2.4289 | 9.42E-07 |
| Cebpb | CCAAT enhancer binding protein beta | 2.5994 | 3.94E-06 |
| Nlrc5 | NLR family CARD domain containing 5 | 2.8141 | 8.39E-06 |
| Parp14 | Poly(ADP-ribose) polymerase family member 14 | 3.4472 | 1.21E-10 |
| Ccrl2 | C-C motif chemokine receptor like 2 | 3.7308 | 1.21E-10 |
| Cebpd | CCAAT enhancer binding protein delta | 3.8089 | 4.75E-08 |
| Serpina3n | Serine peptidase inhibitor, clade A, member 3N | 3.868 | 4.12E-05 |
| Fpr2 | Formyl peptide receptor 2 | 4.0039 | 6.42E-04 |
| Timp1 | TIMP metallopeptidase inhibitor 1 | 4.0855 | 1.13E-04 |
| Madcam1 | Mucosal vascular addressin cell adhesion molecule 1 | 4.1031 | 8.19E-03 |
| S100a9 | S100 calcium binding protein A9 | 4.4308 | 2.29E-04 |
| S100a8 | S100 calcium binding protein A8 | 5.1365 | 3.68E-05 |
| Ccl3 | C-C motif chemokine ligand 3 | 5.2437 | 4.98E-05 |
| Mmp3 | Matrix metallopeptidase 3 | 5.9618 | 1.95E-03 |
| Ccl7 | C-C motif chemokine ligand 7 | 6.004 | 9.85E-06 |
| Ccl4 | C-C motif chemokine ligand 4 | 6.128 | 1.41E-05 |
| Acod1 | Aconitate decarboxylase 1 | 9.0902 | 4.99E-07 |
| Cxcl2 | C-X-C motif chemokine ligand 2 | 9.6908 | 1.61E-07 |
| Saa3 | Serum amyloid A3 | 9.7491 | 2.20E-16 |
| Selp | Selectin P | 10.251 | 1.11E-05 |
| 10-wks D, PBS vs. ND, PBS | | | |
| Genes involved in complement and cell death | | | |
| Nfkb2 | Nuclear factor kappa B subunit 2 | 2.9129 | 2.30E-06 |
| C1ra | Complement 1 receptor alpha | 3.3356 | 1.25E-06 |
| C5ar1 | Complement C5a receptor 1 | 3.7928 | 1.29E-07 |
| Birc3 | Baculoviral IAP repeat containing 3 | 4.0432 | 4.76E-07 |
| Bcl3 | BCL3 transcription coactivator | 4.0973 | 4.05E-06 |
| Ptgs2 | Prostaglandin-endoperoxide synthase 2 | 4.207 | 3.00E-08 |
| Fas | Fas cell surface death receptor | 4.4063 | 9.57E-09 |
| Casp4 | Caspase 4 | 4.5833 | 2.27E-06 |
| Gbp2 | Guanylate binding protein 2 | 4.8146 | 2.86E-09 |
| Gbp5 | Guanylate binding protein 5 | 5.3659 | 1.39E-10 |
| C3 | Complement C3 | 5.8447 | 5.65E-12 |
| Cfb | Complement factor B | 6.7552 | 1.58E-19 |
| Genes involved in inflammation | | | |
| Ccl19 | C-C motif chemokine ligand 19 | 3.2057 | 1.23E-04 |
| Nlrp3 | NLR family pyrin domain containing 3 | 3.2298 | 2.95E-05 |
| Nos2 | Nitric oxide synthase 2 | 3.4351 | 2.01E-04 |
| Ifitm1 | Interferon induced transmembrane protein 1 | 3.4828 | 8.49E-08 |
| Ifit1 | Interferon induced protein with tetratricopeptide repeats 1 | 3.5397 | 2.51E-06 |
| Upp1 | Uridine phosphorylase 1 | 3.8648 | 1.18E-06 |
| Tnf | Tumor necrosis factor | 4.9618 | 6.69E-07 |
| Il1b | Interleukin 1 beta | 5.0603 | 1.85E-05 |
| Il12b | Interleukin 12 beta | 5.2063 | 1.26E-04 |
| Ccl11 | C-C motif chemokine ligand 11 | 5.5157 | 1.43E-07 |
| Ccl5 | C-C motif chemokine ligand 5 | 5.7101 | 2.47E-08 |
| Ccl3 | C-C motif chemokine ligand 3 | 6.3651 | 3.17E-07 |
| Ccl7 | C-C motif chemokine ligand 7 | 6.4002 | 2.27E-06 |
| Il1rn | Interleukin 1 receptor antagonist | 6.886 | 1.95E-07 |
| Cxcl5 | C-X-C motif chemokine ligand 5 | 7.2635 | 1.36E-07 |
| Cxcl10 | C-X-C motif chemokine ligand 10 | 8.6481 | 5.69E-14 |
| Cxcl1 | C-X-C motif chemokine ligand 1 | 8.6703 | 4.36E-12 |
| Ccl2 | C-C motif chemokine ligand 2 | 8.8081 | 2.36E-17 |
| Il6 | Interleukin 6 | 8.9535 | 1.70E-06 |
| Genes involved in vascular and neuronal damage | | | |
| Sox17 | SRY-box transcription factor 17 | -1.62132 | 0.038185365 |
| Tek | TEK receptor tyrosine kinase | -1.41631 | 5.67E-03 |
| Ptprb | Protein tyrosine phosphatase receptor type B | -1.30013 | 9.61E-04 |
| Smox | Spermine oxidase | 1.1431 | 8.58E-08 |
| Rasip1 | Ras interacting protein 1 | 1.4712 | 1.92E-05 |
| Ly6c1 | Lymphocyte antigen 6 family member C1 | 1.6709 | 1.40E-07 |
| Ccn1 | Cellular communication network factor 1 | 2.3991 | 4.28E-05 |
| Cdkn1a | Cyclin dependent kinase inhibitor 1A | 2.5257 | 3.60E-08 |
| Irgm1 | Immunity related GTPase M | 2.5453 | 2.01E-05 |
| Thbs1 | Thrombospondin 1 | 2.5637 | 6.24E-05 |
| Vcam1 | Vascular cell adhesion molecule 1 | 2.8234 | 4.85E-06 |
| Ctla2a | Cytotoxic T lymphocyte-associated protein 2 alpha | 2.831 | 1.23E-04 |
| Itgal | Integrin subunit alpha L | 2.8527 | 2.47E-05 |
| Socs3 | Suppressor of cytokine signaling 3 | 3.2502 | 1.03E-05 |
| Angptl4 | Angiopoietin like 4 | 3.4241 | 1.42E-06 |
| Lrg1 | Leucine rich alpha-2-glycoprotein 1 | 3.9076 | 5.56E-05 |
| Lif | LIF interleukin 6 family cytokine | 4.9705 | 1.41E-06 |
| Tlr2 | Toll like receptor 2 | 5.0234 | 3.17E-10 |
| Icam1 | Intercellular adhesion molecule 1 | 5.5111 | 3.41E-13 |
| Steap4 | STEAP4 metalloreductase | 5.7374 | 7.13E-12 |
| Csf3 | Colony stimulating factor 3 | 9.1345 | 3.10E-10 |
| Selp | Selectin P | 11.12 | 1.63E-06 |
| Genes involved in microglia activation and DR pathogenesis | | | |
| Gpr34 | G protein-coupled receptor 34 | -3.09003 | 1.34E-04 |
| P2ry12 | Purinergic receptor P2Y12 | -1.95073 | 1.90E-03 |
| Rlbp1 | Retinaldehyde binding protein 1 | -1.01031 | 9.02E-03 |
| Irf1 | Interferon regulatory factor 1 | 2.2702 | 9.94E-06 |
| Csf1 | Colony stimulating factor 1 | 2.3311 | 4.54E-06 |
| Ms4a6c | Membrane-spanning 4-domains, subfamily A, member 6C | 2.4375 | 2.08E-04 |
| Hck | HCK proto-oncogene, Src family tyrosine kinase | 2.6063 | 1.49E-04 |
| Cebpb | CCAAT enhancer binding protein beta | 2.8576 | 3.33E-07 |
| Slc15a3 | Solute carrier family 15 member 3 | 3.7785 | 1.69E-06 |
| Msr1 | Macrophage scavenger receptor 1 | 3.8064 | 2.37E-05 |
| Maff | MAF bZIP transcription factor F | 3.9353 | 2.57E-09 |
| Cebpd | CCAAT enhancer binding protein delta | 4.0299 | 9.44E-09 |
| Serpina3n | Serine peptidase inhibitor, clade A, member 3N | 4.0617 | 1.68E-05 |
| Timp1 | TIMP metallopeptidase inhibitor 1 | 4.1083 | 1.18E-04 |
| Fpr2 | Formyl peptide receptor 2 | 5.6673 | 9.14E-08 |
| S100a9 | S100 calcium binding protein A9 | 6.3774 | 9.11E-09 |
| S100a8 | S100 calcium binding protein A8 | 7.1801 | 7.60E-10 |
| Lcn2 | Lipocalin 2 | 7.3584 | 1.58E-19 |
| Slfn4 | Schlafen 4 | 7.5536 | 1.03E-14 |
| Ngp | Neutrophilic Granule Protein | 9.5052 | 1.02E-11 |
| Acod1 | Aconitate decarboxylase 1 | 9.8271 | 5.79E-08 |
| Saa3 | Serum amyloid A3 | 10.731 | 1.58E-19 |
| 4-wks D, rAAV-mFKN vs. 4-wks D, PBS | | | |
| Genes involved in complement and cell death | | | |
| Bag3 | BAG cochaperone 3 | 1.24755 | 0.017685663 |
| Ncf2 | Neutrophil cytosolic factor 2 | 1.96775 | 2.06E-03 |
| Serping1 | Serpin family G member 1 | 2.0274 | 4.86E-03 |
| C1qc | Complement C1q C chain | 2.0489 | 1.04E-03 |
| C1qb | Complement C1q B chain | 2.05307 | 7.15E-04 |
| C1qa | Complement C1q A chain | 2.13918 | 4.77E-04 |
| C4b | Complement C4B | 2.14711 | 0.035143617 |
| C3ar1 | Complement C3a receptor 1 | 2.20153 | 7.81E-06 |
| Ctss | Cathepsin S | 2.25828 | 2.13E-04 |
| Cfi | Complement factor I | 2.60664 | 4.05E-05 |
| Cd5l | CD5 molecule like | 3.0424 | 0.043126955 |
| Cryge | Crystallin gamma E | 6.969357135 | 1.12E-05 |
| Crybb3 | Crystallin beta B3 | 7.962872525 | 5.96E-06 |
| Crygf | Crystallin gamma F | 8.107827111 | 2.30E-06 |
| Cryba4 | Crystallin beta A4 | 8.181474793 | 2.16E-05 |
| Cryaa | Crystallin alpha A | 8.29633506 | 5.51E-05 |
| Crybb1 | Crystallin beta B1 | 8.329248764 | 2.30E-06 |
| Crybb2 | Crystallin beta B2 | 8.450248059 | 1.52E-05 |
| Crygs | Crystallin gamma S | 8.47585516 | 2.16E-05 |
| Cryba2 | Crystallin beta A2 | 8.517711297 | 1.57E-05 |
| Cryba1 | Crystallin beta A1 | 8.864756797 | 9.87E-06 |
| Crygb | Crystallin gamma B | 9.298343942 | 9.15E-07 |
| Crygc | Crystallin gamma C | 9.643160316 | 5.99E-07 |
| Cryga | Crystallin gamma A | 9.818355371 | 1.13E-03 |
| Birc7 | Baculoviral IAP repeat containing 7 | 10.59306404 | 7.56E-04 |
| Genes involved in inflammation | | | |
| Cxcl9 | C-X-C motif chemokine ligand 9 | -3.6598 | 9.25E-03 |
| Cxcl11 | C-X-C motif chemokine ligand 11 | -3.0131 | 0.042287977 |
| Il15 | Interleukin 15 | -1.3964 | 0.047137819 |
| Cxcl13 | C-X-C motif chemokine ligand 13 | 2.41416 | 2.13E-04 |
| Ccl6 | C-C motif chemokine ligand 6 | 3.34556 | 3.50E-03 |
| Hspb1 | Heat shock protein family B (small) member 1 | 3.77668 | 2.13E-04 |
| Genes involved in vascular and neuronal damage | | | |
| Apln | Apelin | -1.4056 | 0.017475225 |
| Hes5 | Hes family bHLH transcription factor 5 | -1.134740401 | 0.017945388 |
| Insm1 | INSM transcriptional repressor 1 | -1.1013 | 5.39E-06 |
| Itgb8 | Integrin subunit beta 8 | 1.0194 | 0.048671934 |
| Akap12 | A-kinase anchoring protein 12 | 1.26036 | 2.31E-03 |
| Ahr | Aryl hydrocarbon receptor | 1.30229 | 3.90E-05 |
| Plxnd1 | Plexin D1 | 1.23105 | 4.72E-04 |
| Rhoj | Ras homolog family member J | 1.31071 | 2.21E-03 |
| Clu | Clusterin | 1.4009 | 2.09E-03 |
| Grik3 | Glutamate ionotropic receptor kainate type subunit 3 | 1.54884 | 2.72E-03 |
| Gadd45b | Growth arrest and DNA damage inducible beta | 2.43458 | 4.30E-04 |
| S100a4 | S100 calcium binding protein A4 | 3.832 | 6.69E-04 |
| Edn2 | Endothelin 2 | 4.37696 | 1.75E-06 |
| Genes involved in microglia activation and DR pathogenesis | | | |
| Vim | Vimentin | 1.22126 | 0.039292571 |
| Csf1r | Colony stimulating factor 1 receptor | 1.29226 | 8.26E-03 |
| Irf8 | Interferon regulatory factor 8 | 1.83771 | 3.36E-03 |
| Cd300lf | CD300 molecule like family member f | 1.8409 | 4.64E-03 |
| Ncf4 | Neutrophil cytosolic factor 4 | 1.84379 | 0.046631511 |
| Itgam | Integrin subunit alpha M | 1.84572 | 0.023091327 |
| Cd38 | CD38 molecule | 1.89704 | 0.032064489 |
| Tmem119 | Transmembrane protein 119 | 1.92981 | 7.96E-03 |
| Ccr5 | C-C motif chemokine receptor 5 | 1.95893 | 0.020272074 |
| Tyrobp | Transmembrane immune signaling adaptor TYROBP | 1.97921 | 2.28E-03 |
| Syk | Spleen associated tyrosine kinase | 2.00751 | 0.023427124 |
| Adgre1 | Adhesion G protein-coupled receptor E1 | 2.02755 | 0.017961424 |
| Slamf9 | SLAM family member 9 | 2.19479 | 0.033223831 |
| Cx3cl1 | C-X3-C motif chemokine ligand 1 (fractalkine) | 2.3159 | 5.64E-04 |
| Gfap | Glial fibrillary acidic protein | 2.33611 | 7.75E-04 |
| Lcn2 | Lipocalin 2 | 2.44772 | 0.030655939 |
| Clec7a | C-type lectin domain containing 7A | 2.46049 | 5.98E-03 |
| Ccl7 | C-C motif chemokine ligand 7 | 3.32142 | 7.47E-03 |
| 4-wks D, rAAV-sFKN vs. 4-wks D, PBS | | | |
| Genes involved in complement and cell death | | | |
| B3gnt5 | UDP-GlcNAc:betaGal beta-1,3-N-acetylglucosaminyltransferase 5 | 3.786529443 | 0.036866852 |
| Serpind1 | Serpin family D member 1 | 4.299166133 | 9.22E-03 |
| Cryab | Crystallin alpha B | 4.8199653 | 4.76E-03 |
| Cryge | Crystallin gamma E | 6.848523619 | 3.59E-05 |
| Wnt7b | Wnt family member 7B | 7.119961952 | 4.84E-04 |
| Crygf | Crystallin gamma F | 7.891549103 | 8.13E-06 |
| Cryba4 | Crystallin beta A4 | 8.216657998 | 4.06E-05 |
| Cryaa | Crystallin alpha A | 8.36025425 | 1.06E-04 |
| Crybb3 | Crystallin beta B3 | 8.455066279 | 1.99E-06 |
| Crybb1 | Crystallin beta B1 | 8.505089773 | 1.99E-06 |
| Crygs | Crystallin gamma S | 8.543226515 | 3.75E-05 |
| Cryba2 | Crystallin beta A2 | 8.65178166 | 2.26E-05 |
| Crybb2 | Crystallin beta B2 | 8.733997832 | 1.39E-05 |
| Lim2 | Lens intrinsic membrane protein 2 | 8.760930566 | 8.13E-06 |
| Cryba1 | Crystallin beta A1 | 8.956989574 | 1.45E-05 |
| Lgsn | Lengsin, lens protein with glutamine synthetase domain | 8.994771581 | 1.36E-07 |
| Cryga | Crystallin gamma A | 9.004050342 | 0.016408504 |
| Crygb | Crystallin gamma B | 9.175390949 | 1.70E-06 |
| Crygc | Crystallin gamma C | 9.665891865 | 7.89E-07 |
| Crygd | Crystallin gamma D | 9.976713628 | 1.36E-07 |
| Birc7 | Baculoviral IAP repeat containing 7 | 10.271979 | 4.41E-03 |
| Genes involved in inflammation | | | |
| Hspb1 | Heat shock protein family B (small) member 1 | 3.526525509 | 2.29E-03 |
| Cd24a | CD24 alpha | 3.541670545 | 0.010710846 |
| Genes involved in vascular and neuronal damage | | | |
| Tnnt2 | Troponin T2, cardiac type | 3.041396898 | 7.15E-04 |
| Slit3 | Slit guidance ligand 3 | 3.247833639 | 0.019189575 |
| S100a4 | S100 calcium binding protein A4 | 3.897927601 | 1.71E-03 |
| 10-wks D, rAAV-sFKN vs. 10-wks D, PBS | | | |
| Genes involved in complement and cell death | | | |
| Birc7 | Baculoviral IAP repeat containing 7 | 6.62051 | 0.030277755 |
| Lgsn | Lengsin, lens protein with glutamine synthetase domain | 7.026691917 | 0.023191617 |
| Crygc | Crystallin gamma C | 7.04664 | 0.049126576 |
| Crygb | Crystallin gamma B | 7.34112 | 0.029567166 |
| Crygd | Crystallin gamma D | 7.42775 | 0.025824189 |
